# Alcohol consumption patterns and association with educational attainment in Europe: an analysis of the 2023-24 European Social Survey

**DOI:** 10.1186/s12889-026-28558-9

**Published:** 2026-07-29

**Authors:** Nour Mahrouseh, Diana A. Grad, Carlos Alexandre Soares Andrade, Sarah Nayani, Jeroen J. A. Spijker, Romana Haneef, Brigid Unim, Mary Economou, Mirza Balaj, Terje Andreas Eikemo, Carl Michael Baravelli, Elena von der Lippe, Grant M. A. Wyper, Orsolya Varga, José Chen-Xu

**Affiliations:** 1 Department of Public Health and Epidemiology, Faculty of Medicine, University of Debrecen, Debrecen, Hungary; 2 Department of Public Health, Faculty of Political, Administrative and Communication Sciences, Babeș-Bolyai University, Cluj-Napoca, Romania; 3 Department of Periodontology, Oro-Dental and Implant Surgery, Faculty of Medicine, University of Liège, CHU of Liège, Liège, Belgium; 4 Department of Epidemiology and Public health, Sciensano, Brussels, Belgium; 5 Institute for Advanced Family Studies (IESF), Universitat Internacional de Catalunya, Barcelona, Spain; 6 Centre d’Estudis Demogràfics, Universitat Autònoma de Barcelona, Bellatarra, Spain; 7 CERPOP, UMR1295, unite mixte INSERM – University of Toulouse III Paul Sabatier, Toulouse, France; 8 Research Unit in Public Health, Epidemiology and Health Economics, University of Liège, Liège, Belgium; 9 Department of Cardiovascular, Endocrine-metabolic Diseases and Aging, Italian National Institute of Health, Rome, Italy; 10 Department of Nursing, School of Health Sciences, Cyprus University of Technology, Limassol, Cyprus; 11 Research Centre for Education and the Labour Market (ROA), Maastricht University, Maastricht, the Netherlands; 12 Centre for Health Equity Analytics (CHAIN), Department of Sociology and Political Science, Norwegian University of Science and Technology (NTNU), Trondheim, Norway; 13 Department of Disease Burden, Norwegian Institute of Public Health, Bergen, Norway; 14 Department of Epidemiology and Health Monitoring, Robert Koch Institute, Berlin, Germany; 15 School of Health and Wellbeing, University of Glasgow, Glasgow, UK; 16 Syreon Research Institute, Budapest, Hungary; 17 Comprehensive Health Research Centre (CHRC), Public Health Research Center, LA-REAL, National School of Public Health, Universidade NOVA de Lisboa, Lisboa, Portugal

**Keywords:** Alcohol consumption, Education, Public health, Risk factor

## Abstract

**Background:**

Alcohol consumption is a risk factor to several non-communicable diseases, posing a threat to vulnerable populations. This study aims to analyse the association between alcohol consumption patterns and educational attainment across Europe.

**Methods:**

a cross-sectional study was conducted using pooled data from the European Social Survey 2023–2024 Round 11, which included individual data from 33,987 respondents aged between 25 and 74 years from 27 European countries. Alcohol consumption was assessed using self-reported frequency and quantity of consumption, using standardised questions to assess consumption patterns and alcohol quantities. Our main outcome was frequency of alcohol consumption. A mixed ordinal logistic regression model assessed associations between sex, age, educational attainment, and alcohol consumption.

**Results:**

The highest percentages of frequent drinkers were found in Western Europe (28.0%) and the lowest in Eastern Europe (14.5%), whereas moderate drinking was most common in Northern Europe (52.5%) and lowest in Eastern Europe (28.9%). The regression model indicated that female gender was positively associated with alcohol consumption (adjOR = 2.05, *p* < 0.001). Primary education was found to be a risk factor compared to tertiary education (adjOR = 1.31, *p* = 0.022). Early adulthood age groups (25–34 and 35–44) presented a positive association compared to the 65–74 group (adjOR = 1.44, *p* = 0.003 and adjOR = 1.33, *p* = 0.001, respectively).

**Conclusions:**

Being female and having lower educational attainment were associated with increased alcohol consumption. The differences in consumption patterns may, in part, reflect the heterogeneity of alcohol policy frameworks across European countries, including variations in taxation, availability restrictions, marketing regulations, and enforcement strategies. Together, these findings highlight that age-specific drinking patterns reflect a combination of socialisation, cultural normalisation, and health-related transitions across the life course.

**Supplementary Information:**

The online version contains supplementary material available at https://doi.org/10.1186/s12889-026-28558-9.

## Introduction

Alcohol consumption is a leading health-harming risk for several preventable non-communicable diseases [1], including different types of cancer [2], cardiovascular diseases [3], and both intentional and unintentional injuries [4]. It implicitly increases disease burden on already strained health systems [5]. In the European Union (EU), the most recent estimates indicate that alcohol-attributable cancer accounted for approximately 80,000 deaths and 1.9 million disability-adjusted life years (DALYs) in 2016 [1]. During the COVID-19 pandemic, mortality rates due to alcohol-specific causes increased across 14 countries, rising by 7.7% and 8.2% for females and males, respectively in 2020, compared with 2019, with the highest increases reported in the Baltic countries [6]. In addition to its health impact, alcohol consumption imposes a substantial economic burden, production losses attributable to alcohol-related mortality in the EU were estimated 32.1 billion euros in 2016, with the highest costs reported in Germany and France [7].

Given the considerable health and economic burden associated with alcohol use, effective policy responses are critical. At the global level, measures to reduce alcohol consumption vary in their effectiveness, with the WHO “Best Buys” recognized as cost-effective strategies [8]. For alcohol, three types of best buys interventions with immediate effects on consumption have been identified: (a) alcohol pricing and taxing; (b) restrictions on alcohol advertising; (c) restrictions on physical availability. How these immediate effects differ by age, sex and socioeconomic background are not yet well understood [9]. Within the EU, implementation remains uneven, as not all countries apply excise duties to the most consumed types of alcohol [10]. Beyond taxation, complementary approaches such as alcohol warning or health warning labels have also been explored. Evidence from a systematic review indicates that their effectiveness depends on factors including message exposure frequency, health literacy, and the frequency of alcohol consumption [11].

Alcohol consumption disproportionately increases vulnerability and susceptibility among specific groups [12], including individuals with mental disorders [13], those with lower educational attainment [14], and people experiencing long-term unemployment [15, 16]. At the same time, alcohol use is also embedded within broader drinking cultures across Europe, including among the working population, where up to 20% report serious alcohol-related problems [17]. Evidence from a study conducted among European university students further highlights the extent of this issue, with problematic alcohol use (CAGE score > 1) reported by 24% of male and 13% of female students [18].

The understanding of alcohol as a health risk factor has evolved significantly over time. Early observational studies suggested that moderate alcohol consumption, particularly red wine, might have cardiovascular benefits, contributing to the notion of a “U” and then “J-shaped curve”, association between alcohol intake and mortality risk [19, 20]. However, more recent and robust evidence has challenged this interpretation. A large meta-analysis involving data from 195 countries concluded that there is no safe level of alcohol consumption, as even low levels were associated with increased risks of cancer and other diseases [21, 22]. Importantly, the health effects of alcohol are not uniform but vary by age, sex, socioeconomic status, genetic susceptibility, baseline health status, drinking patterns, and contextual factors such as consumption with food [23].

Alcohol-related health effects also vary across regions, with socioeconomic and cultural factors playing significant roles as they shape exposure and vulnerability [24]. For example, evidence from Spain shows relative educational inequalities in alcohol-related mortality with age, consistent with cumulative disadvantage over the life course, indicating that populations with lower education attainment bear a disproportionate health burden [25]. Overall, while alcohol use contributes substantially to disease and death, its health impacts are unequally distributed across populations.

Studies have also shown that individuals with higher socioeconomic status tend to drink more frequently, whereas those with lower socioeconomic status are more likely to engage in heavy episodic (or binge) drinking [26–29]. Moreover, sex and education have been reported to be closely interlinked in shaping alcohol consumption patterns. For example, a Danish study found that low education combined with high alcohol consumption significantly increased the risk of alcohol-related medical events, particularly among men [30]. Similarly, research from the United Kingdom showed that heavy adolescent drinking reduced postsecondary educational attainment among working-class males but had little effect on females [31]. This makes alcohol consumption an important public health issue, where cultural norms can sustain high consumption patterns across European countries [32] and social inequalities amplify alcohol-related harm, underscoring the need for policies that address both overall consumption and educational disparities.

Despite existing evidence, alcohol consumption patterns across Europe remain insufficiently characterised, particularly in relation to socioeconomic and demographic factors. Previous analyses, including those based on ESS 2014 data [33], reported that lower education is associated with less frequent drinking but higher engagement in heavy episodic drinking [34]. However, several important gaps remain. First, evidence is limited on how alcohol consumption patterns vary jointly by education, sex, and age across European regions. Second, few studies have explicitly examined regional heterogeneity in these social gradients, despite known cultural and policy differences across Europe. Third, most existing analyses rely on data collected nearly a decade ago, leaving uncertainty about whether previously observed patterns persist under recent social and economic changes. Using data from ESS Round 11 (2023/2024), our study aimed to describe current alcohol consumption patterns in Europe and to examine their associations with age, sex, and education level.

## Methods

### Study design and data sources

This cross-sectional study used data from Round 11 of the European Social Survey (ESS), collected in 2023/2024 [33]. The data releases included respondents from 27 of the 31 participating countries (Austria, Belgium, Bulgaria, Croatia, Cyprus, Finland, France, Germany, Greece, Hungary, Iceland, Ireland, Italy, Lithuania, Latvia, Montenegro, The Netherlands, Norway, Poland, Portugal, Serbia, Slovakia, Slovenia, Spain, Sweden, Switzerland, and the United Kingdom). The ESS, conducted biannually since 2001 by ESS European Research Infrastructure Consortium, investigates attitudes, beliefs and drinking behaviours across Europe using newly selected cross-sectional samples. In Round 11, data were collected primarily via face-to-face video interviews [35, 36].

STROBE Guidelines for observational studies [37] were followed to report our study (Supplementary Table S1).

### Data collection

The data were collected using the ESS source questionnaire. The health module gathers comprehensive data on various aspects of individuals’ physical and mental health. It includes questions on self-rated health, the presence of chronic illnesses or conditions, limitations in daily activities, and recent experiences with healthcare services. Additionally, the survey covers health-related behaviours such as smoking, alcohol consumption, physical activity, and sleep patterns. In some rounds, it also includes items on mental well-being, such as depressive symptoms and emotional support. Questions regarding social inequalities in health and their various determinants, subjective wellbeing and determinants; as well as sociodemographic data (i.e., household composition, sex, age, income, education and occupation) are also included [35].

### Sampling process and eligibility criteria

The ESS adheres to rigorous methodological standards to ensure data comparability across countries and over time [38, 39]. These standards include the use of probability sampling to guarantee representative samples in each participating country. They also require a minimum effective sample size to maintain statistical validity, and a maximum non-response rate of 30% to reduce response bias [40].

The ESS survey population includes all individuals aged 15 years and older who reside in private households within each participating country, regardless of nationality, citizenship, or language. This approach ensures broad representativeness and allows comparisons across diverse populations and survey rounds [40]. This definition is consistently applied across all ESS rounds. For this study, we restricted the main analysis to the participants aged 25 to 74 years old, as those aged 75 years and older may represent a selective group of relatively healthy, non-institutionalised individuals.

### Measurement of alcohol consumption

Alcohol consumption in the ESS questionnaire was assessed across two dimensions: frequency of alcohol consumption and quantity of alcohol consumed. ESS applied conversion factors of different alcohol beverages per country to determine alcohol quantities [41]. For our study, the following key questions were used to record these aspects:

- “*In the last 12 months*,* how often have you had a drink containing alcohol*?” — captured the overall frequency of alcohol consumption (i.e., every day; several times a week; once a week; 2 to 3 times a month; once a month; less than once a month; never; don’t know).
- “*Please think about the last time you were drinking alcohol on a Monday*,* a Tuesday*,* a Wednesday or a Thursday***”** – recorded weekday drinking quantities, calculating a weighted mean alcohol consumption, in grams.
- **“***Now please think about the last time you were drinking alcohol on a Friday*,* a Saturday or a Sunday***”** – recorded weekend drinking quantities, calculating a weighted mean alcohol consumption, in grams.
- “*How many of each of the drinks did you have on that day*?” — recorded the quantity of different types of alcohol consumed in a single drinking session.

The frequency of alcohol consumption was further categorised in frequent drinkers, defined as individuals who report consuming alcohol daily or several times a week, and moderate drinkers, which include those who report consuming alcohol once a week, 2 to 3 times a month, or once a month. Respondents who reported drinking alcohol less than once a month in the last 12 months or never were classified as minimal drinkers [35].

### Statistical analysis

From the retrieved data, we calculated mean alcohol consumption (grams) with respective 95% confidence intervals (CI) based on a binomial distribution, along with prevalence rates of drinking patterns by country and sex. We conducted analyses using regional classifications commonly applied in Eurostat (The European Union’s statistical office), grouping countries into: Central and Eastern; Northern; Southern; and Western Europe. Latvia and Lithuania were included in the Central and Eastern Europe as they present a similar epidemiological profile as this group [42].

We then conducted a mixed-effects ordinal logistic regression model to assess the association between educational attainment, categorised as completing primary (International Standard Classification of Education) (ISCED 0–2), secondary (ISCED 3–4) and tertiary education (ISCED 5–8) [43], and alcohol consumption patterns (minimal, moderate, and frequent drinking), using minimal drinking as the reference category. Country was included as a random intercept to account for between-country variability in drinking behaviours. As research shows that alcohol consumption patterns are associated with an interplay between sex and educational attainment [30], we included an interaction term between these two variables in our analysis and described the interaction term. A sensitivity analysis was also carried without the interaction term, available in the Supplementary Material Table S8. Additionally, given that the questions related to alcohol consumption habits were included once before in the survey, a comparison of ESS Round 11 (2023/24) with ESS Round 7 (2014) was conducted, by calculating percentage differences between the two points. Finally, we carried out marginal effects models to quantify the outcome changes with the shift in alcohol consumption. P-values were interpreted in the context of a 0.05 threshold, but emphasis was placed on effect sizes and confidence intervals. Stata (version 19.0) [44] and R (version 4.4.2) [45] were used to calculate estimates and conduct the regression analyses.

## Results

The survey included 33,987 respondents from 27 European countries: ten from Central and Eastern Europe (Bulgaria, Croatia, Hungary, Montenegro, Latvia, Lithuania, Poland, Serbia, Slovakia, and Slovenia), four from Northern Europe (Finland, Iceland, Sweden, and Norway), five belonging to Southern Europe (Cyprus, Greece, Italy, Portugal, and Spain) and eight from Western Europe (Austria, Belgium, France, Germany, Ireland, Netherlands, Switzerland, and United Kingdom). Table 1 and Supplementary Table S2 provide a breakdown of alcohol consumption habits across variables, disaggregated by educational attainment.

**Table 1:** Prevalence of alcohol consumption habits across Europe and its subregions, by educational attainment

Education Level	Primary			Secondary			Tertiary			Total		
Alcohol frequency/Variables	Frequent Drinker n(%)	Moderate Drinker n(%)	Minimal drinker n(%)	Frequent Drinker n(%)	Moderate Drinker n(%)	Minimal drinker n(%)	Frequent Drinker n(%)	Moderate Drinker n(%)	Minimal drinker n(%)	Frequent Drinker n(%)	Moderate Drinker n(%)	Minimal drinker n(%)
Total	2442 (22.01)	3454 (31.13)	5201 (46.87)	2317 (18.82)	4461 (36.23)	5536 (44.96)	2339 (22.12)	4579 (43.30)	3658 (34.59)	7098 (21.0)	12,494 (36.9)	14,395 (42.1)
Sex												
Male	1880 (34.28)	1803 (32.87)	1802 (32.85)	1697 (28.97)	2342 (39.99)	1818 (31.04)	1472 (31.53)	2012 (43.10)	1184 (25.36)	5049 (31.6)	6157(38.7)	4804 (29.8)
Female	562 (10.01)	1651 (29.42)	3399 (60.57)	620 (9.60)	2119 (32.82)	3718 (57.58)	867 (14.68)	2567 (43.45)	2474 (41.88)	2049 (11.4)	6337 (35.2)	9591 (53.3)
Age group												
25–34	170 (16.49)	404 (39.19)	457 (44.33)	273 (14.35)	806 (42.38)	823 (43.27)	346 (15.20)	1085 (47.65)	846 (37.15)	789 (15.3)	2295 (43.1)	2126 (41.6)
35–44	269 (18.13)	537 (36.19)	678 (45.69)	395 (16.58)	928 (38.96)	1059 (44.46)	491 (18.08)	1218 (44.85)	1007 (37.08)	1155 (17.6)	2683 (40.0)	2744 (42.4)
45–54	492 (23.10)	672 (31.55)	966 (45.35)	518 (18.60)	1047 (37.59)	1220 (43.81)	524 (22.06)	1108 (46.65)	743 (31.28)	1534 (21.3)	2827 (38.6)	2929 (40.1)
55–64	703 (23.72)	959 (32.35)	1302 (43.93)	609 (20.90)	1011 (34.69)	1294 (44.41)	517 (28.95)	706 (39.53)	563 (31.52)	1829 (24.5)	2676 (35.5)	3159 (40.0)
65–74	808 (23.17)	882 (25.29)	1798 (51.55)	522 (22.39)	669 (28.70)	1140 (48.91)	461 (32.42)	462 (32.49)	499 (35.09)	1791 (26.0)	2013 (28.8)	3437 (45.2)
Regions												
East	562 (16.45)	823 (24.09)	2,031 (59.46)	755 (14.23)	1,494 (28.15)	3,058 (57.62)	398 (12.89)	1,094 (35.44)	1,595 (51.67)	1715 (14.5)	3411 (28.9)	6684 (56.6)
North	81 (13.21)	289 (47.15)	243 (39.64)	190 (14.86)	643 (50.27)	446 (34.87)	336 (18.52)	1,014 (55.90)	464 (25.58)	607 (16.4)	1946 (52.5)	1153 (31.1)
South	661 (25.66)	615 (23.87)	1,300 (50.47)	575 (21.00)	1,052 (38.42)	1,111 (40.58)	402 (20.82)	839 (43.45)	690 (35.73)	1638 (22.6)	2506 (34.6)	3101 (42.8)
West	1,138 (25.33)	1,727 (38.45)	1,627 (36.22)	797 (26.66)	1,272 (42.54)	921 (30.80)	1,203 (32.13)	1,632 (43.59)	909 (24.28)	3138 (28.0)	4631 (41.3)	3457 (30.8)

Frequent drinking was most frequently reported in Western Europe (28.0%), and least frequently in Eastern Europe (14.5%). Moderate drinking was most frequently reported in Northern Europe (52.5%) and least frequently in Eastern Europe (28.9%). Minimal drinking was most frequently reported in Eastern Europe (56.6%) and least frequently reported in Western (30.8%) and Northern Europe (31.1%). Across age groups, frequent and minimal drinking were most prevalent in the 65–74 age group (26.0% and 45.2%, respectively), whereas moderate drinking was most prevalent in the 25–34 age group (43.1%).

Prevalence of minimal drinkers was consistently higher in females, representing more than half of the group (53.3%). On the other hand, males present high rates of frequent and moderate drinkers (31.6% and 38.7%, respectively), with varying frequencies depending on the education level.

Individuals with tertiary education reported higher prevalence of frequent and moderate drinking (22.1% and 43.3%, respectively), whereas minimal drinking was more prevalent among individuals with primary education (46.9%). The prevalence of minimal drinking was similar across regions, while moderate drinking was more prevalent than frequent drinking in all regions.

Alcohol consumption across European countries and European subregions was described using the prevalence of each consumption pattern (Supplementary Table S3), and the weighted mean of alcohol consumption (Tables 2 and 3, and Supplementary Tables S4 and S5).

Eastern Europe showed the highest mean alcohol consumption among frequent drinkers in weekdays across all three education categories, reaching to 65.68 g (48.06–83.30) (Table 2). Weekday consumption among frequent drinkers in the other three regions was relatively similar. During weekends, Northern Europe showed the highest consumption among individuals with primary education (112.57 g, 71.41-153.74), whereas Eastern Europe recorded the highest consumption among those with secondary and tertiary education (78.35 g and 64.97 g, respectively). Among moderate drinkers, alcohol consumption patterns are similar, with Eastern Europe presenting the highest mean consumption across all regions and all educational groups. With exception of frequent male drinkers in Eastern Europe, males with tertiary education showed lower average weekday alcohol consumption than those with primary or secondary education across all regions. Among females, with the exception of moderate weekday drinkers in Southern Europe, no educational gradient in alcohol consumption was observable across regions.

The countries with the highest percentages of frequent drinkers (Supplementary Table S3) were the Netherlands (32.4%) and Belgium (31.5%), whereas moderate drinking was more prevalent in Norway (53.0%), Sweden (51.8%), and Finland (48.9%). Conversely, the countries with the lowest percentages of frequent drinkers were Lithuania (6.7%), Poland (7.0%), and Hungary (8.5%), and those with lowest rates of moderate drinkers were Hungary (20.4%), Portugal (21.6%), and Italy (24.2%).

**Fig. 1:**
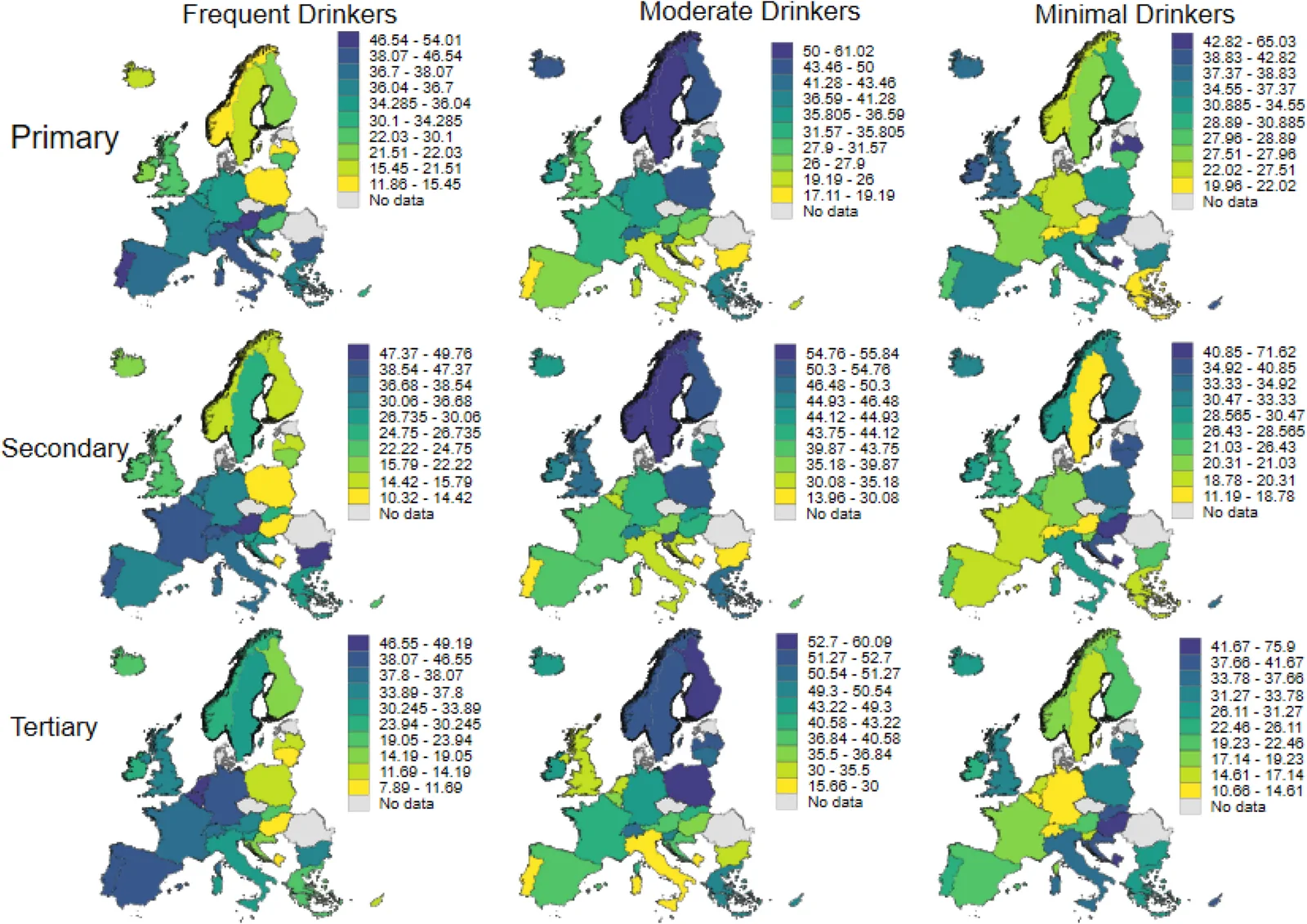
Prevalence (%) of alcohol consumption patterns by educational attainment level for each country for males

**Fig. 2:**
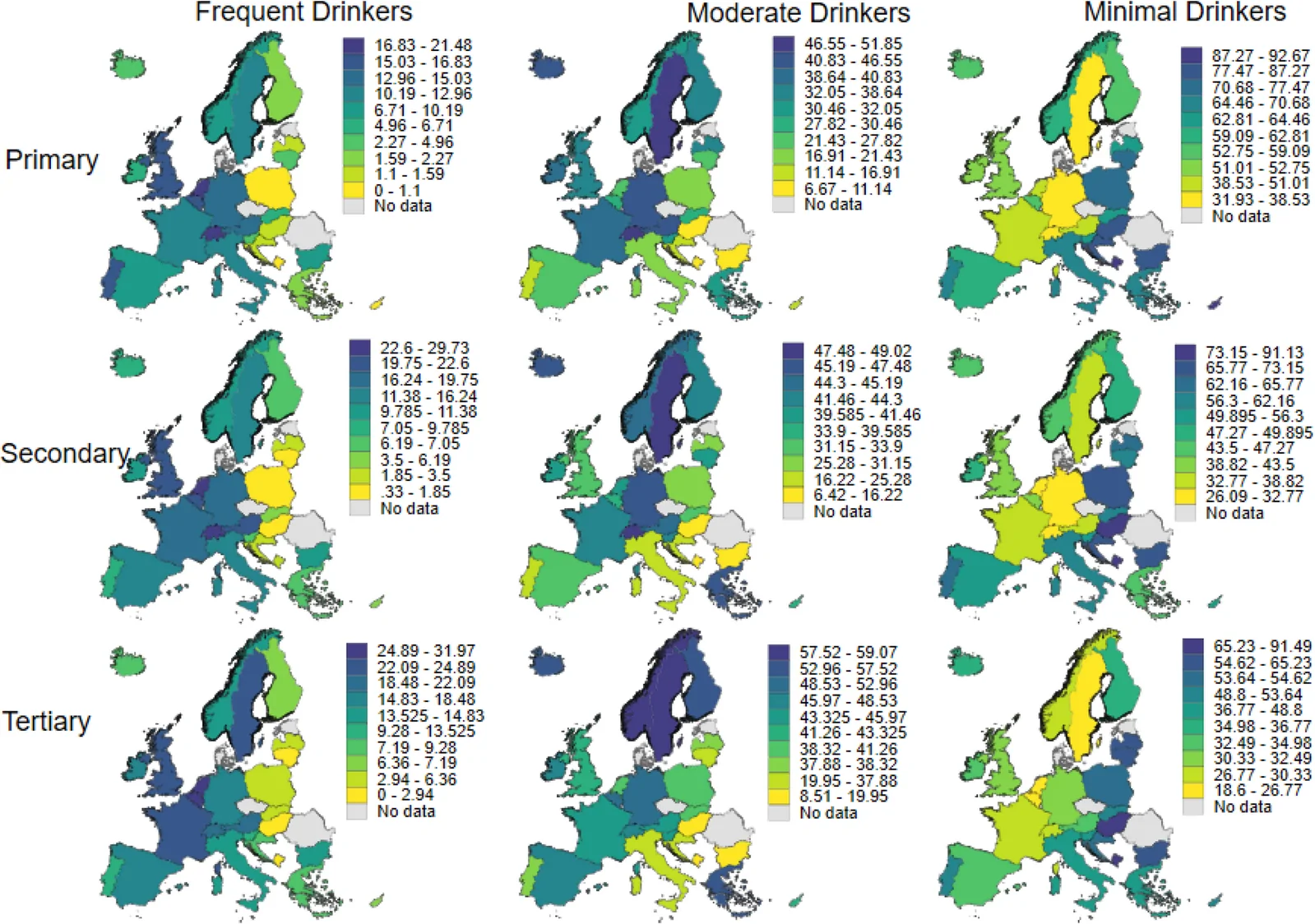
Prevalence (%) of alcohol consumption patterns by educational attainment level for each country for females

Figures 1 and 2 represent the prevalence of different alcohol consumption patterns across European countries by sex and education level. Males show a higher prevalence of drinking (both frequent and moderate) across all education levels than females, who were more likely to be minimal drinkers in most countries. However, this gap narrows at higher education levels, as females with more education show higher drinking prevalence. The highest percentages of frequent drinking among males were observed in Portugal (54.0%), Austria (48.5%) and Italy (46.5%) for primary education; Bulgaria (49.8%), Austria (48.7%) and Portugal (47.4%) for secondary education; and the Netherlands (49.2%), Belgium (49.1%) and Portugal (46.6%) for tertiary education.

A trend analysis of alcohol consumption based on two European Social Surveys (2014 and 2023/24) showed a 20.0% decrease in the share of individuals with primary education who reported frequent drinking, while this share increased among people with secondary (17.0%) and tertiary education (8.0%). A similar pattern was observed amongst moderate drinkers, with 21% reduction among those with primary education and an increase of 4.0% and 29.0% among those with secondary and tertiary education, respectively. The percentage of minimal drinkers increased in all groups. Detailed information is presented in Supplementary Table S6.

**Table 2:** Weighted Mean of alcohol consumption per grams for weekdays of frequent and moderate drinkers by educational attainment level, sex and region

Alcohol consumption habits for weekdays									
**Region**	**Frequent Drinker** **Male**	**Frequent Drinker** **Female**	**Frequent Drinker** **Both**	**Moderate Drinker** **Male**	**Moderate Drinker** **Female**	**Moderate Drinker** **Both**	**Minimal drinker** **Male**	**Minimal drinker** **Female**	**Minimal drinker** **Both**
Education Level	Primary								
East	65.14 (55.14–75.15)	50.91 (30.24–71.58)	64.03 (54.64–73.42)	60.54 (44.15–76.93)	24.95 (19.21–30.69)	50.67 (38.50–62.85)	12.59 (3.82–21.36)	5.42 (2.33–8.52)	8.04 (4.28–11.80)
North	39.20 (24.77–53.63)	27.16 (18.72–35.60)	37.20 (25.02–49.37)	28.98 (21.63–36.33)	19.34 (14.52–24.15)	25.62 (20.49–30.75)	7.94 (2.83–13.05)	2.79 (1.50–4.08)	5.05 (2.70–7.41)
South	42.54 (36.47–48.61)	25.61 (20.71–30.50)	38.92 (33.99–43.85)	22.42 (18.42–26.42)	22.02 (13.42–30.63)	22.24 (17.76–26.72)	2.63 (1.33–3.93)	1.74 (1.08–2.41)	2.04 (1.42–2.66)
West	39.07 (34.75–43.39)	30.00 (24.74–35.26)	36.36 (32.92–39.81)	21.37 (18.62–24.13)	12.83 (10.98–14.68)	16.68 (15.02–18.34)	4.96 (1.16–8.75)	3.38 (2.37–4.39)	3.98 (2.41–5.55)
Education Level	Secondary								
East	69.73 (48.72–90.73)	46.66 (30.27–63.04)	65.68 (48.06–83.30)	53.86 (41.63–66.09)	28.54 (22.59–34.50)	43.68 (35.83–51.52)	14.16 (7.12–21.20)	11.87 (3.91–19.82)	12.60 (6.74–18.46)
North	39.05 (31.13–46.97)	40.21 (21.29–59.12)	39.41 (31.39–47.43)	31.07 (24.71–37.43)	23.65 (19.24–28.06)	28.07 (23.86–32.28)	13.03 (7.42–18.64)	7.35 (4.87–9.82)	9.79 (6.97–12.62)
South	43.71 (36.78–50.65)	38.17 (28.73–47.61)	42.24 (36.56–47.92)	22.24 (18.47–26.02)	15.08 (11.66–18.49)	19.16 (16.54–21.78)	3.92 (1.98–5.86)	2.54 (1.80–3.28)	2.97 (2.18–3.76)
West	38.40 (33.44–43.36)	27.25 (22.95–31.56)	34.33 (30.80–37.87)	24.40 (20.73–28.07)	16.14 (12.41–19.87)	20.06 (17.43–22.69)	4.60 (3.08–6.12)	3.55 (2.28–4.82)	3.93 (2.96–4.91)
Education Level	Tertiary								
East	73.88 (47.53–100.23)	39.34 (25.72–52.96)	62.14 (43.71–80.57)	47.15 (30.64–63.65)	31.52 (16.49–46.55)	39.31 (28.13–50.48)	18.46 (4.27–32.65)	5.89 (3.14–8.64)	9.69 (4.89–14.49)
North	35.99 (30.64–41.33)	26.00 (21.48–30.52)	31.63 (27.94–35.32)	27.32 (23.65–31.00)	18.79 (16.73–20.85)	22.27 (20.32–24.23)	11.30 (5.99–16.60)	7.84 (5.75–9.93)	9.03 (6.75–11.30)
South	31.58 (27.07–36.09)	29.34 (17.33–41.34)	30.77 (25.59–35.96)	16.65 (13.64–19.66)	11.96 (9.76–14.15)	13.73 (11.96–15.51)	3.19 (1.41–4.97)	3.44 (2.00–4.88)	3.36 (2.23–4.49)
West	33.34 (30.32–36.37)	30.78 (26.73–34.83)	32.40 (29.97–34.83)	20.14 (17.19–23.09)	17.02 (14.39–19.65)	18.39 (16.43–20.35)	4.20 (2.92–5.47)	4.41 (3.00–5.82)	4.33 (3.33–5.33)

**Table 3:** Weighted Mean of alcohol consumption per grams for weekends of frequent and moderate drinkers by educational attainment level, sex and region

Alcohol consumption habits for weekends									
**Region**	**Frequent Drinker** **Male**	**Frequent Drinker** **Female**	**Frequent Drinker** **Both**	**Moderate Drinker** **Male**	**Moderate Drinker** **Female**	**Moderate Drinker** **Both**	**Minimal drinker** **Male**	**Minimal drinker** **Female**	**Minimal drinker** **Both**
Education Level	Primary								
East	82.72 (67.49–97.96)	53.83 (33.73–73.94)	80.46 (66.28–94.63)	91.14 (69.89–112.38)	42.41 (25.62–59.20)	77.63 (61.36–93.90)	25.09 (13.78–36.41)	9.54 (4.59–14.49)	15.21 (9.97–20.46)
North	127.30 (81.32–173.27)	38.82 (29.14–48.50)	112.57 (71.41–153.74)	70.35 (58.02–82.68)	36.74 (31.30–42.18)	58.64 (49.98–67.31)	16.58 (9.05–24.10)	14.63 (10.09–19.16)	15.48 (11.33–19.64)
South	52.79 (45.81–59.78)	32.46 (27.30–37.63)	48.44 (42.77–54.12)	48.03 (39.97–56.10)	27.77 (23.24–32.31)	38.82 (33.85–43.80)	4.62 (2.91–6.32)	3.86 (2.55–5.16)	4.11 (3.08–5.15)
West	51.37 (46.63–56.10)	42.66 (36.05–49.27)	48.77 (44.90–52.63)	42.77 (36.17–49.38)	31.56 (26.18–36.95)	36.62 (32.38–40.86)	7.77 (5.04–10.51)	6.74 (5.43–8.05)	7.13 (5.82–8.45)
Education Level	Secondary								
East	82.70 (71.51–93.89)	57.90 (40.36–75.44)	78.35 (68.58–88.13)	86.78 (70.93–102.63)	45.86 (35.96–55.76)	70.32 (59.76–80.87)	23.30 (14.67–31.94)	16.91 (8.44–25.37)	18.95 (12.57–25.34)
North	66.88 (57.16–76.61)	47.17 (39.53–54.80)	60.77 (53.52–68.01)	65.07 (57.92–72.23)	43.58 (38.93–48.24)	56.39 (51.63–61.15)	40.22 (29.74–50.69)	17.46 (13.67–21.25)	27.26 (22.08–32.43)
South	54.07 (48.75–59.39)	48.63 (38.04–59.22)	52.62 (47.80–57.44)	39.89 (35.03–44.74)	28.15 (24.72–31.58)	34.83 (31.64–38.03)	7.93 (4.80–11.06)	9.66 (0.12–19.21)	9.13 (2.47–15.78)
West	60.47 (53.16–67.77)	41.26 (33.60–48.92)	53.46 (47.99–58.92)	45.19 (39.56–50.82)	36.58 (28.74–44.41)	40.67 (35.76–45.57)	11.71 (8.53–14.89)	9.31 (6.49–12.13)	10.18 (8.05–12.32)
Education Level	Tertiary								
East	72.70 (58.84–86.56)	49.96 (39.04–60.87)	64.97 (54.78–75.16)	81.51 (58.24–104.78)	46.74 (34.77–58.72)	64.07 (50.81–77.32)	15.98 (12.03–19.94)	11.99 (9.70–14.28)	13.20 (11.20–15.20)
North	63.84 (56.77–70.92)	40.75 (35.87–45.64)	53.77 (49.01–58.53)	60.48 (53.62–67.33)	45.06 (37.33–52.78)	51.35 (45.95–56.74)	21.73 (15.68–27.77)	22.28 (17.39–27.18)	22.09 (18.27–25.92)
South	47.79 (42.59–52.99)	44.56 (31.84–57.29)	46.63 (41.00–52.27)	33.08 (29.47–36.69)	26.50 (24.10–28.90)	28.99 (26.96–31.03)	8.05 (5.10–11.00)	5.79 (3.58–8.01)	6.54 (4.77–8.32)
West	56.51 (51.01–62.00)	49.72 (43.41–56.02)	53.99 (49.82–58.17)	44.06 (39.64–48.48)	31.78 (28.71–34.86)	37.18 (34.49–39.87)	7.59 (5.22–9.96)	8.24 (6.01–10.47)	7.99 (6.34–9.64)

### Association between alcohol consumption and socioeconomic factors

Females with primary education had higher odds of alcohol consumption compared to males with tertiary education (adjusted odds ratio [adjOR] = 1.75, 95%CI 1.25 to 2.45), as did females with secondary education (adjOR = 1.38, 95%CI 1.06 to 1.80). Age was modestly positively associated with alcohol consumption, with higher odds observed among individuals aged 25–34 years (adjOR = 1.44, 95%CI 1.13 to 1.84) and 35–44 years (adjOR = 1.33, 95%CI 1.11 to 1.58), compared with those aged 65–74 years. Regional sub-analysis confirmed broadly similar patterns in Northern and Southern Europe, whereas in Western and Eastern Europe the estimated sex-differences within education groups were smaller and less clear (Table 4). Sex-stratified sub-analysis showed similar results (Supplementary Table S7).

**Table 4:** Mixed-effects ordinal logistic regression model showing the association of frequency of alcohol consumption and socioeconomic variables, for aggregated 27 countries and per European subregions

Variables	Adjusted OR (95%CI)	*p* value
European-wide		
Female sex (ref: male sex)	2.05 (1.63–2.59)	< 0.001
Age		
25–34	1.44 (1.13–1.84)	0.003
35–44	1.33 (1.11–1.58)	0.001
45–54	1.14 (0.90–1.43)	0.283
55–64	0.99 (0.83–1.18)	0.92
65–74 (ref)	1	
Education		
Primary	1.31 (1.04–1.64)	0.022
Secondary	1.08 (0.94–1.24)	0.278
Tertiary (ref)	1	
Sex: education		
Female and Primary	1.75 (1.26–2.45)	0.001
Female and Secondary	1.38 (1.06–1.80)	0.018
Female and Tertiary (ref)	1	
Eastern Europe		
Female sex (ref: male sex)	2.65 (2.28–3.07)	< 0.001
Age		
25–34	0.73 (0.57–0.93)	0.012
35–44	0.71 (0.62–0.81)	< 0.001
45–54	0.57 (0.42–0.78)	< 0.001
55–64	0.72 (0.66–0.79)	< 0.001
65–74 (ref)	1	
Education		
Primary	0.84 (0.65–1.07)	0.16
Secondary	0.92 (0.71–1.19)	0.528
Tertiary (ref)	1	
Sex: education		
Female and Primary	2.66 (2.09–3.38)	< 0.001
Female and Secondary	1.65 (1.28–2.15)	< 0.001
Female and Tertiary (ref)	1	
Northern Europe		
Female sex (ref: male sex)	1.75 (1.38–2.23)	< 0.001
Age		
25–34	2.76 (1.64–4.64)	< 0.001
35–44	2.13 (1.20–3.77)	0.01
45–54	1.22 (0.83–1.79)	0.317
55–64	1.15 (0.71–1.88)	0.566
65–74 (ref)	1	
Education		
Primary	1.90 (1.50–2.42)	< 0.001
Secondary	1.74 (1.36–2.22)	< 0.001
Tertiary (ref)	1	
Sex: education		
Female and Primary	2.27 (2.11–2.45)	< 0.001
Female and Secondary	1.25 (1.00–1.56)	0.049
Female and Tertiary (ref)	1	
Southern Europe		
Female sex (ref: male sex)	2.11 (1.81–2.45)	< 0.001
Age		
25–34	1.04 (0.80–1.36)	0.75
35–44	1.16 (0.89–1.51)	0.262
45–54	0.91 (0.81–1.02)	0.107
55–64	0.80 (0.57–1.13)	0.202
65–74 (ref)	1	
Education		
Primary	1.04 (0.61–1.76)	0.896
Secondary	0.95 (0.75–1.21)	0.667
Tertiary (ref)	1	
Sex:education		
Female and Primary	2.44 (1.60–3.71)	< 0.001
Female and Secondary	1.78 (1.48–2.14)	< 0.001
Female and Tertiary (ref)	1	
Western Europe		
Female sex (ref: male sex)	1.97 (1.31–2.96)	0.001
Age		
25–34	1.94 (1.70–2.23)	< 0.001
35–44	1.65 (1.46–1.87)	< 0.001
45–54	1.56 (1.38–1.77)	< 0.001
55–64	1.21 (1.06–1.38)	0.004
65–74 (ref)	1	
Education		
Primary	1.67 (1.51–1.85)	< 0.001
Secondary	1.20 (1.03–1.41)	0.022
Tertiary (ref)	1	
Sex:education		
Female and Primary	1.30 (0.97–1.74)	0.077
Female and Secondary	1.11 (0.75–1.63)	0.615
Female and Tertiary (ref)	1	

A negative marginal effect predicted mean for females indicates that females have a significantly lower predicted probability of being frequent alcohol drinkers compared with males across all countries (Figure S1; Supplementary Table S9). The marginal effects ranged between (− 0.163 to − 0.143), indicating that being female is associated with a 14–16% point lower probability of frequent alcohol consumption relative to males.

Individuals aged 25–34 and 35–44 show significantly negative marginal effects across studied countries, indicating a lower probability of frequent alcohol consumption relative to the oldest reference group of 65–74 (Figure S2). The age groups 45–54 and 55–64 do not differ significantly from the 65–74 group in any country. Younger age groups are less likely to be frequent drinkers compared to the oldest age group. Individuals with primary education have a substantially lower predicted probability of frequent alcohol consumption across all countries (approximately − 0.07) compared to the reference category of having tertiary education (Figure S3). Those with secondary education also have a significantly lower probability to be frequent drinker, though the marginal predicted mean is smaller around − 0.03 compared to those with higher education (tertiary).

## Discussion

Across 33,987 adults in 27 countries, frequent drinking was most prevalent in Western Europe and least common in Eastern Europe, whereas moderate drinking was most common in Northern Europe and lowest in Eastern Europe. By age and sex, both frequent and moderate use peaked at 45–64 years, while non-drinking at 65–74 years. Females were more likely to be minimal drinkers, whereas males were more often frequent or moderate drinkers, although the magnitude of these differences varied across regions and educational levels. Mean intake also varied by region and education: Eastern Europe showed the highest weekday consumption among frequent drinkers across all education levels; on weekends, Northern Europe led among those with primary education, while Eastern Europe led among secondary and tertiary education groups. Males consistently consumed more alcohol than females, and moderate drinkers reported lower overall intake compared with frequent drinkers. Country-level patterns differed: frequent drinking was most common in the Netherlands and Belgium, while moderate drinking was most prevalent in Norway, Sweden, and Finland. Lithuania, Poland, and Hungary had the lowest levels of frequent drinking, and moderate drinking was least common in Hungary, Portugal, and Italy. Mediterranean countries showed mixed patterns, with higher moderate consumption in Spain and lower levels in Italy.

In adjusted models, alcohol consumption varied by age, and with the interaction of sex and education. Marginal contrasts indicated that females with lower education had higher odds overall, when compared to those with tertiary education. At the European level, younger age groups (higher odds at 25–44) had higher odds of alcohol consumption than those aged 65–74 years, whereas in Eastern Europe lower age was associated with lower odds. The sex-education interaction was not observed in the Western or Eastern European regions.

### Regional differences

Alcohol consumption patterns were not uniform across Europe. Regional sociocultural contexts may influence the relationship between education and alcohol consumption. Significant interactions between sex and education were observed in Eastern, Southern, and Northern Europe, indicating lower education may be associated with higher drinking patterns. However, the interaction was not significant in Western Europe, indicating no effect of education on women in the western region. Differences in regions were also observed in the association between alcohol drinking patterns and age groups. Western Europe showed young groups may drink more than older age groups, which is the contrary in Eastern region, as the model showed that older age group 65–74 have higher drinking patterns compared to younger group, the quantity of alcohol consumed among frequent drinkers was generally higher in Eastern Europe. Previous studies have similarly reported higher levels of alcohol consumption in Eastern Europe, where spirits have traditionally been the predominant alcoholic beverage, whereas beer consumption is more common in Central and Western Europe. These differences may partly explain the variation observed between European regions in our analyses [46–48].

### Age differences

Alcohol consumption patterns in Europe exhibit a clear age-related gradient, consistent with other international evidence [34, 49]. Moderate drinking was found to be most common among younger adults (25–34 years), likely reflecting social drinking patterns and greater participation in nightlife, and peer-related activities [50]. In contrast, frequent drinking peaks in older age, particularly between 55 and 74 years, which may reflect more routine and culturally embedded drinking habits, such as daily wine or beer consumption with meals or after [51]. At the same time, non-drinking is also most prevalent in the 65–74 age group, suggesting a polarization of behaviours in later life between sustained frequent drinkers and those who abstain altogether [49, 50], possibly due to health concerns or medical advice. Similar age-related patterns have been reported elsewhere [52, 53].

The regression models were consistent with these descriptive patterns, showing higher reported alcohol consumption among younger adults (25–44) compared with older age groups, although the differences narrowed with age, which may hint to the possibility of occurring a gradual shift from social to habitual consumption. Regionally, these trends are broadly consistent, but the strength of the age gradient varied. Consumption was particularly pronounced in Northern Europe, where younger cohorts reported higher consumption, and less marked in Eastern Europe where older adults continued to report substantial drinking volumes. Another study noted that older adults tended to report better health than minimal drinkers as less healthy former heavy drinkers move to lower drinking categories [54]. These differences may, in part, reflect the heterogeneity of alcohol policy frameworks across European countries, including variations in taxation, availability restrictions, marketing regulations, and enforcement strategies [55, 56]. Together, these findings highlight that age-specific drinking patterns reflect a combination of socialisation, cultural norms, and health-related transitions across the life course [57].

### Sex and education

This study further contributes to a growing body of evidence on the relationship between education and alcohol consumption in Europe, especially as it intersects with sex. Our results show regional variation in the education-alcohol gradient, with higher consumption risk among those with lower education most evident in Northern and Western Europe. However, this pattern was not consistent across Europe, suggesting that social, economic, and cultural factors shape these associations differently across Europe [58].

Our results also show notable sex-education differences, with relatively higher odds of more frequent alcohol consumption among primary-educated females, especially in Eastern and Southern Europe. These patterns differ from earlier evidence based on ESS 2014 data, which found lower education associated with less frequent but more heavy episodic drinking [58]. In contrast, our more recent data suggest a shift in drinking patterns, with lower-educated females appearing to have higher levels of frequent alcohol consumption, as captured by drinking frequency rather than episodic drinking. These changes may hint to the possibility of broader social transitions taking place, including economic insecurity and changing cultural norms around female drinking [59–61].

Prevalence patterns further support this shift. Among males, frequent drinking was most prevalent among those with primary education, whereas moderate drinking was more common among those with secondary and tertiary education. For females, non-drinking was more common among those with lower educational attainment, while moderate drinking was highest among tertiary education. These findings are consistent with Kilian et al. (2023), who reported higher prevalence rates among males and females with high socioeconomic status for past-year alcohol use. However, their study also showed that risky single occasion drinking was more prevalent among females with high SES, suggesting the need to distinguish between consumption frequency and episodic drinking patterns [58] when examining socioeconomic inequalities in alcohol use.

Huijts et al. also reported that individuals with lower education were generally less likely to be frequent drinkers in most Northern and Western European countries, but this association was less consistent in Central/Eastern Europe [34]. Our results suggest a clearer association between lower educational attainment and frequent drinking, particularly among females, suggesting possible temporal changes in social determinants of alcohol use.

Another study of ESS Round 7 data showed a positive educational gradient for frequent alcohol consumption, with higher predicted probabilities among individuals with high education, while patterns of heavy episodic drinking showed different associations with education [62]. In particular, earlier ESS-based studies reported heavy episodic drinking to be more common among individuals with lower or middle education. Although higher education has generally been associated with less heavy episodic drinking, more recent findings indicate a complex relationship between education and drinking patterns, influenced by factors such as financial stress, health knowledge, and social norms [62, 63]. Differences in alcohol policies, including pricing and availability, as well as cultural contexts, may further contribute to variation in observed drinking patterns across populations [50, 64, 65].

Our study contributes to the existing literature employing ESS data to quantify sex and educational inequalities through its methodological approach to geographical units of analysis. It is an addition to previous studies that have examined the association between education and alcohol consumption (as well as other risky behaviours) in 21 European countries with subregional analysis [34], by expanding the number of countries analysed and the inclusion of the interaction between sex and education.

Overall, differences in alcohol drinking patterns may be subjected to many factors. Previous studies have reported that survey results of alcohol consumption patterns are under-reported. Survey results capture only 30 to 60% of the true alcohol sales [66]. Under-reporting alcohol drinking may rise from recall bias; particularly among heavy drinkers and individuals with irregular drinking patterns [67], as well as from social desirability bias and cultural norms surrounding alcohol use [68]. Underreporting may also vary by age and sex [69], several studies suggesting that younger adults underestimate their alcohol consumption more than older adults [70, 71]. The results of a study carried out in Australia, comparing the results of alcohol of two surveys (National Drug Strategy Household Survey (NDSHS) and the more detailed International Alcohol Control (IAC) survey) showed that overall consumption was underestimated by approximately 33% in the National survey. The magnitude of underestimation was more pronounced among young males (40%) and middle-aged females (49%) and less shown among young females (15%) and older females [72].

In our study, men show higher absolute prevalence of drinking patterns across educational groups. However, females demonstrate higher relative gradient across educational groups. Higher odds of women does not denote higher alcohol consumption compared to men; but rather stronger association with drinking patterns. As women have lower prevalence of alcohol consumption at baseline, this may translate into larger relative differences, resulting in higher odds ratios. Our findings therefore highlight inequalities in alcohol consumption among women while acknowledging that men continue to account for a larger share of overall alcohol consumption.

### Strengths and limitations

This study examines alcohol consumption patterns in 27 European countries. The use of individual level data allowed assessing drinking patterns according to sex and education level of the respondents. The ESS has implemented equivalent instruments in all the countries and survey waves, which enables analytical comparisons over time and across geographical regions. In addition, the use of probability sampling in the surveys ensures that the results are nationally representative.

However, there are several limitations that need to be considered when interpreting the results. Previous research has shown that survey data may not fully capture total adult alcohol consumption, leading to potential underestimation, that varies across countries [73]. Additionally, the face-to-face interviews may also have resulted in underreporting of the true alcohol consumption of the respondents, due to social desirability bias [74]. Furthermore, the measurement of the overall frequency of alcohol consumption requires a recall for the last 12 months, which may introduce recall bias. While we analysed consumption patterns as reported by the ESS, we did not conduct further analysis on heavy episodic drinking. Finally, as this analysis is the cross-sectional analysis, it identifies association between drinking patterns and education level but cannot infer causality.

### Implications for policy and practice

The findings underscore the importance of understanding how regional, educational, and sociodemographic factors shape alcohol consumption in Europe. Future research should examine these variations in greater depth, including potential shifts in drinking patterns among females and younger adults, as well as the association of consumption and episodic drinking with employment status. From a policy perspective, responses should be tailored to regional and national contexts, with effects of policies varying by socioeconomic group [75]. In Northern Europe, where alcohol taxation and availability are already tightly regulated, further reductions in alcohol consumption may benefit more from targeted prevention strategies, including community-based interventions and initiatives addressing drinking norms among younger adults [76, 77]. In Eastern Europe, where previous research has documented frequent drinking is less common, but higher volumes consumed, policies should prioritise reducing harmful consumption through minimum unit pricing or increases in alcohol taxation, regulation of informal alcohol markets, and improved access to counselling and treatment for high-risk drinkers [78, 79]. Western Europe faces the challenge of normalising daily drinking, which could be addressed by stricter advertising regulations, clearer labelling of alcohol content, and promotion of alcohol-free alternatives in social and professional settings [11, 80–83]. In Southern Europe, moderate drinking is culturally entrenched, yet preventive measures remain necessary to avoid escalation into risky behaviours among youth, including school-based education, parental awareness campaigns, and limits on youth-targeted alcohol marketing [76, 84]. Across all regions, males and younger adults remain key targets for intervention, but trends among females require continued monitoring to inform gender-sensitive strategies. Education also emerges as an important determinant, highlighting the need for prevention programs tailored to different educational groups, addressing both quantities among lower-educated populations and the normalisation of frequent consumption among the highly educated.

These findings should therefore be interpreted as evidence on self-reported drinking patterns rather than as a direct measure of alcohol-related harm. Survey-based estimates are valuable for identifying sociodemographic and regional differences in consumption, but they may underestimate alcohol use and cannot fully capture downstream outcomes such as alcohol-attributable mortality, morbidity, injuries, or social harms. Consequently, policy decisions should not rely on survey-based consumption indicators alone, but should interpret them alongside mortality-based indicators, hospitalisation data, and other measures of alcohol-related harm.

## Conclusion

Overall, our findings emphasise changing patterns in alcohol consumption in Europe, with increasing odds among primary-educated females. This highlights the need to combine universal measures such as taxation, labelling, and advertising restrictions with region- and population-specific interventions. Continuous monitoring and research are critical to ensure that policies remain effective and responsive to evolving drinking patterns across Europe.

## Supplementary Information

Supplementary Material 1.

## Data Availability

All data supporting the findings of this study are available within the paper and its Supplementary Information. The complete dataset is openly available at https://ess.sikt.no/.
