# Supplementary material for: Alcohol consumption patterns and association with educational attainment in Europe: an analysis of the 2023-24 European Social Survey
Source: BMC Public Health. 2026 Jul 29;26:3017. doi: 10.1186/s12889-026-28558-9 (PMC13681755; doi:10.1186/s12889-026-28558-9)
Supplement: Supplementary file 1 [file 12889_2026_28558_MOESM1_ESM.docx]

**Supplementary Material**

Mahrouseh et al 2026

*Alcohol consumption patterns and association with educational attainment in Europe: an analysis of the 2023-24 European Social Survey*

Table S1. STROBE Statement—checklist of items that should be included in reports of observational studies

|  | Item No | Recommendation | Location |
| --- | --- | --- | --- |
| **Title and abstract** | 1 | (*a*) Indicate the study’s design with a commonly used term in the title or the abstract | Page 1 |
|  |  | (*b*) Provide in the abstract an informative and balanced summary of what was done and what was found | Page 3 |
| Introduction | | |  |
| Background/rationale | 2 | Explain the scientific background and rationale for the investigation being reported | Page 4 |
| Objectives | 3 | State specific objectives, including any prespecified hypotheses | Page 5 Para 3 |
| Methods | | |  |
| Study design | 4 | Present key elements of study design early in the paper | Page 5 Para 4 |
| Setting | 5 | Describe the setting, locations, and relevant dates, including periods of recruitment, exposure, follow-up, and data collection | Page 5 Para 4 |
| Participants | 6 | (*a*) *Cohort study*—Give the eligibility criteria, and the sources and methods of selection of participants. Describe methods of follow-up  *Case-control study*—Give the eligibility criteria, and the sources and methods of case ascertainment and control selection. Give the rationale for the choice of cases and controls  *Cross-sectional study*—Give the eligibility criteria, and the sources and methods of selection of participants | Page 6 Para 2-3 |
|  |  | (*b*) *Cohort study*—For matched studies, give matching criteria and number of exposed and unexposed  *Case-control study*—For matched studies, give matching criteria and the number of controls per case | NA |
| Variables | 7 | Clearly define all outcomes, exposures, predictors, potential confounders, and effect modifiers. Give diagnostic criteria, if applicable | Page 6 Para 4-5 |
| Data sources/ measurement | 8* | For each variable of interest, give sources of data and details of methods of assessment (measurement). Describe comparability of assessment methods if there is more than one group | Para 6 Para 4-5; Page 7 Para 3 |
| Bias | 9 | Describe any efforts to address potential sources of bias | Page 7 Para 3 |
| Study size | 10 | Explain how the study size was arrived at | Page 5 Para 4 |
| Quantitative variables | 11 | Explain how quantitative variables were handled in the analyses. If applicable, describe which groupings were chosen and why | Page 7 Para 2 |
| Statistical methods | 12 | (*a*) Describe all statistical methods, including those used to control for confounding | Page 7 Para 3 |
|  |  | (*b*) Describe any methods used to examine subgroups and interactions | Page 7 Para 3 |
|  |  | (*c*) Explain how missing data were addressed | NA |
|  |  | (*d*) *Cross-sectional study*—If applicable, describe analytical methods taking account of sampling strategy | Page 6 Para 3 |
|  |  | (*e*) Describe any sensitivity analyses | Page 7 Para 3 |

| Results | | |  |
| --- | --- | --- | --- |
| Participants | 13* | (a) Report numbers of individuals at each stage of study—eg numbers potentially eligible, examined for eligibility, confirmed eligible, included in the study, completing follow-up, and analysed | Page 7 Para 4 |
|  |  | (b) Give reasons for non-participation at each stage | NA |
|  |  | (c) Consider use of a flow diagram | NA |
| Descriptive data | 14* | (a) Give characteristics of study participants (eg demographic, clinical, social) and information on exposures and potential confounders | Page 7 Para 4; Table 1 |
|  |  | (b) Indicate number of participants with missing data for each variable of interest | NA |
|  |  | (c) *Cohort study*—Summarise follow-up time (eg, average and total amount) | NA |
| Outcome data | 15* | *Cohort study*—Report numbers of outcome events or summary measures over time | *NA* |
|  |  | *Case-control study—*Report numbers in each exposure category, or summary measures of exposure | *NA* |
|  |  | *Cross-sectional study—*Report numbers of outcome events or summary measures | Page 9 Para 1 |
| Main results | 16 | (*a*) Give unadjusted estimates and, if applicable, confounder-adjusted estimates and their precision (eg, 95% confidence interval). Make clear which confounders were adjusted for and why they were included | Page 14 Table 3 |
|  |  | (*b*) Report category boundaries when continuous variables were categorized | NA |
|  |  | (*c*) If relevant, consider translating estimates of relative risk into absolute risk for a meaningful time period | NA |
| Other analyses | 17 | Report other analyses done—eg analyses of subgroups and interactions, and sensitivity analyses | Page 16 Para 2 |
| Discussion | | |  |
| Key results | 18 | Summarise key results with reference to study objectives | Page 16 Para 3 |
| Limitations | 19 | Discuss limitations of the study, taking into account sources of potential bias or imprecision. Discuss both direction and magnitude of any potential bias | Page 18 Para 6 |
| Interpretation | 20 | Give a cautious overall interpretation of results considering objectives, limitations, multiplicity of analyses, results from similar studies, and other relevant evidence | Page 17 Para 3 |
| Generalisability | 21 | Discuss the generalisability (external validity) of the study results | Page 19 Para 3 |
| Other information | | |  |
| Funding | 22 | Give the source of funding and the role of the funders for the present study and, if applicable, for the original study on which the present article is based | Page 2 |

Table S2 - Distribution of frequency of alcohol consumption per education level and European regions.

| **Education Level** | **Primary** | | | | | |
| --- | --- | --- | --- | --- | --- | --- |
| **Alcohol Frequency** | Frequent Drinkers | Moderate Drinkers | Minimal Drinkers | Frequent Drinkers | Moderate Drinkers | Minimal drinkers |
| **Region/Gender** | Male | Male | Male | Female | Female | Female |
| **East** | 517 (29.0%) | 720 (40.5%) | 543 (30.5%) | 280 (17.1%) | 45 (2.8%) | 1311 (80.1%) |
| **North** | 64 (17.5%) | 112 (30.6%) | 190 (51.9%) | 99 (40.1%) | 17 (6.9%) | 131 (53.0%) |
| **South** | 524 (42.8%) | 378 (30.9%) | 323 (26.4%) | 292 (21.6%) | 137 (10.1%) | 922 (68.3%) |
| **West** | 775 (36.7%) | 592 (28.0%) | 747 (35.3%) | 980 (41.2%) | 363 (15.3%) | 1035 (43.5%) |
| **Total** | 1880 (34.3%) | 1802 (32.9%) | 1803 (32.9%) | 1651 (29.4%) | 562 (10.0%) | 3399 (60.6%) |
| **Education Level** | **Secondary** | | | | | |
| **Alcohol Frequency** | Frequent Drinkers | Moderate Drinkers | Minimal Drinkers | Frequent Drinkers | Moderate Drinkers | Minimal drinkers |
| **Region/Gender** | Male | Male | Male | Female | Female | Female |
| **East** | 621 (25.5%) | 995 (40.8%) | 822 (33.7%) | 672 (23.4%) | 134 (4.7%) | 2063 (71.9%) |
| **North** | 138 (18.9%) | 200 (27.4%) | 392 (53.7%) | 251 (45.7%) | 52 (9.5%) | 246 (44.8%) |
| **South** | 432 (34.7%) | 300 (24.1%) | 513 (41.2%) | 539 (36.1%) | 143 (9.6%) | 811 (54.3%) |
| **West** | 506 (35.0%) | 323 (22.4%) | 615 (42.6%) | 657 (42.5%) | 291 (18.8%) | 598 (38.7%) |
| **Total** | 1697 (29.0%) | 1818 (31.0%) | 2342 (40.0%) | 2119 (32.8%) | 620 (9.6%) | 3718 (57.6%) |
| **Education Level** | **Tertiary** | | | | | |
| **Alcohol Frequency** | Frequent Drinkers | Moderate Drinkers | Minimal Drinkers | Frequent Drinkers | Moderate Drinkers | Minimal drinkers |
| **Region/Gender** | Male | Male | Male | Female | Female | Female |
| **East** | 255 (21.1%) | 446 (36.8%) | 510 (42.1%) | 584 (31.1%) | 143 (7.6%) | 1149 (61.3%) |
| **North** | 204 (25.7%) | 162 (20.4%) | 429 (54.0%) | 585 (57.4%) | 132 (13.0%) | 302 (29.6%) |
| **South** | 270 (31.8%) | 239 (28.1%) | 341 (40.1%) | 498 (46.1%) | 132 (12.2%) | 451 (41.7%) |
| **West** | 743 (41.0%) | 337 (18.6%) | 732 (40.4%) | 900 (46.6%) | 460 (23.8%) | 572 (29.6%) |
| **Total** | 1472 (31.5%) | 1184 (25.4%) | 2012 (43.1%) | 2567 (43.5%) | 867 (14.7%) | 2474 (41.9%) |

Table S3. Drinking habits by gender and country.

| **Country** | **Males** |  |  | **Females** |  |  | **Total** | | |
| --- | --- | --- | --- | --- | --- | --- | --- | --- | --- |
|  | Frequent drinkers | Moderate drinkers | Minimal Drinkers | Frequent drinkers | Moderate drinkers | Minimal Drinkers | Frequent Drinkers | Moderate drinkers | Minimal Drinkers |
|  | N(%) | N(%) | N(%) | N(%) | N(%) | N(%) | N(%) | N(%) | N(%) |
| **AT** | 447(45.11) | 334(33.70) | 210(21.19) | 235(17.28) | 573(42.13) | 552(40.59) | 682(29.01) | 907(38.58) | 762(32.41) |
| **BE** | 321(39.73) | 281(34.78) | 206(25.50) | 180(22.93) | 322(41.02) | 283(36.05) | 501(31.45) | 603(37.85) | 483(30.70) |
| **CH** | 266(38.33) | 311(31.38) | 117(11.81) | 136(19.83) | 341(49.71) | 209(30.47) | 402(29.13) | 652(47.25) | 326(23.62) |
| **CY** | 70(22.80) | 117(38.11) | 120(39.09) | 19(5.08) | 119(31.82) | 236(63.10) | 89(13.07) | 236(34.65) | 356(52.28) |
| **DE** | 443(36.58) | 485(40.05) | 283(28.56) | 196(16.28) | 555(46.10) | 453(37.62) | 639(26.46) | 1040(43.06) | 736(30.48) |
| **ES** | 311(35.54) | 280(32.00) | 284(32.46) | 123(12.71) | 327(33.78) | 518(53.51) | 434(23.55) | 607(32.94) | 802(43.52) |
| **FI** | 133(17.34) | 399(52.02) | 235(30.64) | 49(6.18) | 364(45.90) | 380(47.92) | 182(11.67) | 763(48.91) | 615(39.42) |
| **FR** | 307(35.33) | 333(38.32) | 229(26.35) | 145(16.18) | 367(40.96) | 384(42.86) | 452(25.61) | 700(39.66) | 613(34.73) |
| **GB** | 264(32.20) | 279(34.02) | 277(33.78) | 176(20.63) | 302(35.40) | 375(43.96) | 440(26.30) | 581(34.73) | 652(38.97) |
| **GR** | 346(27.97) | 588(47.53) | 303(24.49) | 97(6.43) | 629(41.68) | 783(51.89) | 443(16.13) | 1217(44.32) | 1086(39.55) |
| **HR** | 200(28.17) | 216(30.42) | 294(41.41) | 30(3.53) | 202(23.74) | 619(72.74) | 230(14.73) | 418(26.78) | 913(58.49) |
| **HU** | 157(18.92) | 278(33.49) | 395(47.59) | 22(1.73) | 152(11.92) | 1101(86.35) | 179(8.50) | 430(20.43) | 1496(71.07) |
| **IE** | 204(22.59) | 359(39.76) | 340(37.65) | 130(11.74) | 441(39.84) | 536(48.42) | 334(16.62) | 800(39.80) | 876(43.58) |
| **IS** | 76(18.23) | 192(46.04) | 149(35.73) | 34(8.02) | 196(46.23) | 194(45.75) | 110(13.08) | 388(46.14) | 343(40.78) |
| **IT** | 514(39.06) | 353(26.82) | 449(34.12) | 180(11.94) | 331(21.96) | 996(66.09) | 694(24.58) | 684(24.23) | 1445(51.19) |
| **LT** | 75(14.42) | 238(45.77) | 207(39.81) | 16(1.92) | 273(32.69) | 546(65.39) | 91(6.72) | 511(37.71) | 753(55.57) |
| **NL** | 337(40.07) | 296(35.20) | 208(24.73) | 212(24.88) | 315(36.97) | 325(38.15) | 549(32.43) | 611(36.09) | 533(31.48) |
| **NO** | 132(19.61) | 369(54.83) | 172(25.56) | 77(11.60) | 339(51.05) | 248(37.35) | 209(15.63) | 708(52.95) | 420(31.41) |
| **PL** | 82(12.37) | 323(48.72) | 258(38.91) | 17(2.25) | 240(31.83) | 497(65.92) | 99(6.99) | 563(39.73) | 755(53.28) |
| **PT** | 287(49.65) | 124(21.45) | 167(28.89) | 102(12.83) | 172(21.64) | 521(65.53) | 389(28.33) | 296(21.56) | 688(50.11) |
| **RS** | 210(28.859 | 225(30.91) | 293(40.25) | 65(7.85) | 177(21.38) | 586(70.77) | 275(17.67) | 402(25.84) | 879(56.49) |
| **SE** | 166(25.98) | 333(52.11) | 140(21.91) | 103(17.52) | 303(51.53) | 182(30.95 | 269(21.92) | 636(51.83) | 322(26.24) |
| **SI** | 162(26.69) | 281(46.29) | 164(27.02) | 52(8.15) | 243(38.09) | 343(53.76) | 214(17.19) | 524(42.09) | 507(40.72) |
| **SK** | 180(27.19) | 248(37.46) | 234(35.35) | 44(5.75) | 249(32.55) | 472(61.70) | 224(15.70) | 497(34.83) | 706(49.47) |

Table S4. Mean alcohol consumption per grams for weekdays and weekends of frequent drinkers by gender and country

| **Country** | **Weekdays** | | | **Weekends** | | |
| --- | --- | --- | --- | --- | --- | --- |
|  | Males | Females | Totals | Males | Females | Totals |
|  | mean  (95%CI) | mean  (95%CI) | mean  (95%CI) | mean  (95%CI) | mean  (95%CI) | mean  (95%CI) |
| **AT** | 39.69(36.10-43.28) | 31.29(26.91-35.66) | 39.28(31.48-47.08) | 58.55(55.01-62.08) | 55.06(47.78-62.35) | 57.35(53.93-60.76) |
| **BE** | 42.36(36.07-48.64) | 36.46(30.13-42.78) | 45.09(34.37-55.81) | 59.84(52.01-67.67) | 47.68(39.87-55.49) | 55.48(49.71-61.25) |
| **CH** | 28.64(25.60-31.69) | 22.07(18.72-25.41) | 24.79(21.11-28.47) | 45.48(40.80-50.17) | 35.89(29.20-42.58) | 42.21(38.35-46.07) |
| **CY** | 48.48(39.03-57.93) | 35.18(12.44-57.91) | 45.31(31.58-59.04) | 62.83(50.79-74.87) | 35.47(24.67-46.28) | 57.48(47.34-67.64) |
| **DE** | 32.03(29.65-34.40) | 23.35(20.53-26.18) | 33.75(29.30-38.20) | 47.83(44.11-51.56) | 32.54(29.04-36.04) | 43.18(40.32-46.03) |
| **ES** | 26.77(23.46-30.08) | 22.58(18.06-27.10) | 29.69(25.22-34.16) | 44.60 (40.41-48.79) | 40.24(28.44-52.05) | 43.36(38.87-47.86) |
| **FI** | 40.02(30.97-49.07) | 29.84(18.24-41.44) | 30.09(21.11-39.07) | 62.46(52.12-72.80) | 41.63(32.61-50.65) | 56.82(48.79-64.85) |
| **FR** | 29.19(25.46-30.08) | 20.52(17.05-23.98) | 30.68(24.58-36.77) | 40.61(36.40-44.82) | 32.94(26.41-39.48) | 38.15(34.59-41.70) |
| **GB** | 50.27(44.20-56.33) | 43.19(36.66-49.72) | 57.53(46.07-68.98) | 71.76(64.47-79.04) | 59.31(51.12-67.51) | 66.77(61.28-72.25) |
| **GR** | 51.01(40.10-61.92) | 31.51(22.23-40.78) | 57.05(26.92-87.18) | 64.46(57.38-71.54) | 43.85(35.26-52.43) | 59.95(54.06-65.84) |
| **HR** | 52.40(44.79-60.01) | 22.18(15.80-28.56) | 52.17(42.15-62.19) | 70.16(52.51-87.81) | 25.31(16.98-33.64) | 64.38(48.85-79.91) |
| **HU** | 54.74(41.77-67.72) | 36.29(13.90-58.67) | 64.41(46.12-82.71) | 62.05(54.19-69.91) | 56.45(39.45-73.45) | 61.34(54.16-68.52) |
| **IE** | 58.58(50.98-66.18) | 36.42(30.82-42.02) | 51.11(41.67-60.54) | 90.23(78.47-101.99) | 57.84(51.38-64.30) | 77.59(69.81-85.36) |
| **IS** | 41.55(33.65-49.45) | 26.06(20.39-31.74) | 54.86(33.36-76.35) | 71.75(60.71-82.79) | 49.81(40.23-59.40) | 65.19(56.74-73.63) |
| **IT** | 44.72(40.50-48.94) | 38.65(30.21-47.09) | 45.17(39.78-50.56) | 52.73(48.68-56.77) | 41.67(34.78-48.55) | 49.86(46.36-53.36) |
| **LT** | 132.42(94.41-170.44) | 59.14(31.33-86.95) | 217.62(60.90-374.33) | 157.61(123.58-191.64) | 84.00(52.61-115.39) | 145.63(116.17-175.08) |
| **NL** | 26.64(23.53-29.75) | 18.81(16.34-21.27) | 24.71(21.66-27.75) | 49.84(43.90-55.78) | 32.67(29.02-36.33) | 43.20(39.23-47.17) |
| **NO** | 33.04(28.61-37.46) | 27.13(21.87-32.39) | 33.10(9.97-56.23) | 66.18(58.33-74.04) | 42.83(36.21-49.45) | 57.68(51.94-63.42) |
| **PL** | 75.41(50.03-100.78) | 43.76(14.33-73.20) | 109.71(43.31-176.12) | 33.71(30.78-36.64) | 54.71(34.22-75.19) | 75.62(60.44-90.80) |
| **PT** | 28.23(25.49-30.97) | 16.75(14.84-18.67) | 26.77(23.92-29.62) | 80.98(67.54-94.41) | 20.46(17.06-23.86) | 30.25(27.84-32.66) |
| **RS** | 65.16(52.41-77.90) | 37.28(27.14-47.42) | 58.64(42.90-74.38) | 62.43(53.90-70.97) | 48.64(31.20-66.09) | 73.64(62.42-84.86) |
| **SE** | 35.30(30.08-40.52) | 27.05(20.96-33.14) | 35.97(27.89-44.04) | 45.72(39.09-52.34) | 39.06(34.58-43.54) | 53.59(47.86-59.32) |
| **SI** | 43.01(36.73-49.28) | 22.46(15.87-29.06) | 34.82(27.74-41.90) | 45.72(39.09-52.34) | 30.19(18.61-41.77) | 41.95(36.14-47.75) |
| **SK** | 68.21(60.28-76.14) | 67.02(36.12-97.93) | 52.88(40.078-65.68) | 88.74(75.24-102.24) | 67.75(45.25-90.25) | 84.62(72.88-96.36) |

Table S5. Mean alcohol consumption per grams disaggregated for weekdays and weekends of moderate drinkers by gender and country

| **Country** | **Weekdays** | | | **Weekends** | | |
| --- | --- | --- | --- | --- | --- | --- |
|  | Males | Females | Totals | Males | Females | Totals |
|  | mean(95%CI) | mean(95%CI) | mean(95%CI) | mean(95%CI) | mean(95%CI) | mean(95%CI) |
| **AT** | 29.54(24.89-34.19) | 22.29 (19.97-24.61) | 24.98 (22.71-27.25) | 46.01(41.14-50.88) | 45.47(39.97-50.96) | 45.67 (41.77-49.57) |
| **BE** | 26.17(21.78-30.56) | 18.59 (15.14-22.03) | 22.13(19.37-24.90) | 45.58(39.82-51.33) | 40.87(35.53-46.20) | 43.07(39.15-46.98) |
| **CH** | 17.12(15.15-19.09) | 14.19 (12.27-16.11) | 15.59(14.21-16.97) | 33.69(30.15-37.22) | 27.13(23.49-30.78) | 30.28(27.72-32.83) |
| **CY** | 23.44(17.93-28.96) | 22.04 (15.53-28.56) | 22.73 (18.46-27.00) | 33.47(25.01-41.93) | 30.86(25.58-36.15) | 32.14 (27.20-37.09) |
| **DE** | 19.43 (17.45-21.40) | 13.67(12.36-14.98) | 16.36(15.19-17.53) | 37.48(34.09-40.88) | 30.32(24.60-36.05) | 33.65 (30.20-37.11) |
| **ES** | 12.93 (10.46-15.41) | 10.20(6.16-14.23) | 11.46 (9.00-13.91) | 35.89(32.14-39.64) | 24.09(21.88-26.30) | 29.52 (27.37-31.67) |
| **FI** | 26.73 (23.22-30.23) | 17.06 (14.43-19.69) | 22.13 (19.88-24.38) | 67.79(60.96-74.62) | 44.51(31.71-57.31) | 56.70 (49.59-63.81) |
| **FR** | 15.50 (13.22-17.77) | 9.02 (7.80-10.24) | 12.09 (10.82-13.37) | 30.16(26.96-33.35) | 19.22(17.54-20.90) | 24.44 (22.64-26.25) |
| **GB** | 36.63 (31.00-42.26) | 24.41 (20.03-28.79) | 30.31(26.74-33.88) | 57.41(50.53-64.28) | 45.41(39.87-50.94) | 51.23(46.82-55.64) |
| **GR** | 23.95 (21.78-26.12) | 18.30 (16.61-19.99) | 21.03(19.65-22.40) | 40.27(37.84-42.70) | 34.27(29.67-38.86) | 37.17 (34.51-39.82) |
| **HR** | 35.32 (27.78-42.87) | 19.06 (14.20-23.93) | 27.54(22.91-32.17) | 54.07 (43.33-64.81) | 28.77(25.27-32.27) | 41.68(35.81-47.54) |
| **HU** | 31.03 (27.39-34.66) | 22.44(18.26-26.62) | 27.95(25.15-30.75) | 59.33 (51.94-66.73) | 40.28(33.60-46.96) | 52.58 (47.18-57.97) |
| **IE** | 30.33(25.61-35.06) | 19.47(16.38-22.56) | 24.33(21.59-27.07) | 77.14 (71.45-82.83) | 52.96(48.92-56.99) | 63.79 (60.31-67.28) |
| **IS** | 25.10 (20.96-29.25) | 20.00(17.54-22.46) | 22.50(20.10-24.89) | 56.11(49.68-62.54) | 47.05(42.85-51.25) | 51.47(47.65-55.30) |
| **IT** | 27.69 (23.81-31.58) | 23.15(18.61-27.71) | 25.50(22.52-28.48) | 44.18 (38.42-49.93) | 31.18(27.38-34.97) | 37.89(34.37-41.41) |
| **LT** | 65.31 (52.33-78.28) | 36.05(27.59-44.51) | 49.67(42.02-57.32) | 125.82(105.69-145.96) | 61.87(52.69-71.04) | 91.51(80.60-102.41) |
| **NL** | 15.05 (11.65-18.45) | 9.51 (7.66-11,36) | 12.20(10.28-14.11) | 39.08 (34.74-43.43) | 24.57 (21.55-27.59) | 31.61(28.93-34.29) |
| **NO** | 28.93 (25.22-32.64) | 20.84 (18.62-23.06) | 25.05 (22.83-27.28) | 63.70 (58.04-69.36) | 47.10 (42.78-51.42) | 55.74 (52.09-59.39) |
| **PL** | 56.44 (45.33-67.55) | 32.91 (23.25-42.56) | 46.45 (38.80-54.10) | 94.25 (79.09-109.42) | 46.91(35.92-57.90) | 74.16 (64.08-84.24) |
| **PT** | 16.33 (11.21-21.45) | 16.73 (12.93-20.53) | 16.56 (13.50-19.63) | 34.40 (26.94-41.87) | 23.07(18.95-27.19) | 27.84 (23.85-31.83) |
| **RS** | 37.03 (29.59-44.48) | 25.66 (19.18-32.14) | 31.94 (26.88-37.00) | 57.93 (47.38-68.47) | 40.01 (31.70-48.31) | 50.03 (43.04-57.02) |
| **SE** | 25.99 (21.58-30.40) | 19.89 (17.58-22.20) | 23.05 (20.50-25.61) | 56.18 (49.90-62.45) | 37.98(34.99-40.96) | 47.50 (43.85-51,14) |
| **SI** | 24.26 (20.93-27.59) | 15.53(12.86-18.21) | 20.20 (17.99-22.40) | 39.53(33.81-45.25) | 23.33 (19.47-27.19) | 32.09 (28.46-35.72) |
| **SK** | 62.44(54.46-70.43) | 37.85(31.46-44.24) | 50.14 (44.92-55.37) | 83.87(74.43-93.31) | 45.78(41.55-50.00) | 64.79 (59.36-70.21) |

Table S6. Percentage change in alcohol consumption patterns from ESS 2014 and 2023.

| Educational attainment | Frequent Drinker | | | Moderate Drinker | | | Minimal Drinker | | |
| --- | --- | --- | --- | --- | --- | --- | --- | --- | --- |
|  | both | male | female | both | male | female | both | male | female |
| primary | -0.20 | -0.12 | -0.38 | -0.21 | -0.22 | -0.22 | 0.09 | 0.11 | 0.08 |
| secondary | 0.17 | 0.27 | -0.03 | 0.04 | 0.12 | -0.04 | 0.58 | 0.64 | 0.55 |
| Tertiary | 0.08 | 0.10 | 0.05 | 0.29 | 0.31 | 0.27 | 0.67 | 0.84 | 0.60 |

Table S7. Mix multinomial regression models with disaggregation by sex.

|  |  | Male |  |  |  | Female |  |
| --- | --- | --- | --- | --- | --- | --- | --- |
| Region | **Variable** | **OR (95% CI)** | **p-value** | **Region** | **Variable** | **OR (95% CI)** | **p-value** |
| European-wide | 25-34 | 1.483 (1.192 to 1.845) | <0.001 | **European-wide** | 25-34 | 1.397 (1.030 to 1.895) | 0.032 |
|  | 35-44 | 1.349 (1.156 to 1.574) | <0.001 |  | 35-44 | 1.317 (1.041 to 1.665) | 0.021 |
|  | 45-54 | 1.182 (0.948 to 1.473) | 0.138 |  | 45-54 | 1.083 (0.826 to 1.419) | 0.565 |
|  | 55-64 | 0.999 (0.847 to 1.179) | 0.994 |  | 55-64 | 0.973 (0.756 to 1.251) | 0.828 |
|  | 65-74 (reference) | 1 |  |  | 65-74 (reference) | 1 |  |
|  | Primary | 1.371 (1.137 to 1.653) | 0.001 |  | Primary | 2.304 (1.936 to 2.741) | <0.001 |
|  | Secondary | 1.140 (1.023 to 1.272) | 0.018 |  | Secondary | 1.458 (1.234 to 1.722) | <0.001 |
|  | Tertiary (reference) | 1 |  |  | Tertiary (reference) | 1 |  |
| Eastern | 25-34 | 0.975 (0.757 to 1.189) | 0.647 | **Eastern** | 25-34 | 0.511 (0.356 to 0.732) | <0.001 |
|  | 35-44 | 0.846 (0.718 to 0.997) | 0.046 |  | 35-44 | 0.553 (0.480 to 0.637) | <0.001 |
|  | 45-54 | 0.683 (0.487 to 0.959) | 0.028 |  | 45-54 | 0.444 (0.320 to 0.615) | <0.001 |
|  | 55-64 | 0.742 (0.650 to 0.848) | <0.001 |  | 55-64 | 0.665 (0.606 to 0.729) | <0.001 |
|  | 65 to 74 (reference) | 1 |  |  | 65 -74 (reference) | 1 |  |
|  | Primary | 0.881 (0.696 to 1.115) | 0.291 |  | Primary | 2.102 (1.593 to 2.774) | <0.001 |
|  | Secondary | 0.974 (0.758 to 1.252) | 0.839 |  | Secondary | 1.413 (1.206 to 1.657) | <0.001 |
|  | Tertiary (reference) | 1 |  |  | Tertiary (reference) | 1 |  |
| Northern | 25-34 | 2.080 (1.482 to 2.919) | <0.001 | **Northern** | 25-34 | 3.908 (1.754 to 8.707) | 0.001 |
|  | 35-44 | 1.588 (0.958 to 2.631) | 0.073 |  | 35-44 | 2.983 (1.512 to 5.886) | 0.002 |
|  | 45-54 | 1.024 (0.731 to 1.435) | 0.891 |  | 45-54 | 1.500 (0.911 to 2.468) | 0.111 |
|  | 55-64 | 1.036 (0.571 to 1.878) | 0.908 |  | 55-64 | 1.320 (0.799 to 2.180) | 0.278 |
|  | 65-74 (reference) | 1 |  |  | 65-74 (reference) | 1 |  |
|  | primary | 1.732 (1.345 to 2.231) | <0.001 |  | primary | 5.227 (4.090 to 6.680) | <0.001 |
|  | Secondary | 1.667 (1.345 to 2.066) | <0.001 |  | secondary | 2.332 (1.868 to 2.911) | <0.001 |
|  | Tertiary (reference) | 1 |  |  | Tertiary (reference) | 1 |  |
| Southern | 25-34 | 1.155 (0.825 to 1.617) | 0.4 | **Southern** | 25-34 | 0.903 (0.682 to 1.196) | 0.476 |
|  | 35-44 | 1.382 (0.986 to 1.937) | 0.06 |  | 35-44 | 0.924 (0.742 to 1.151) | 0.481 |
|  | 45-54 | 1.025 (0.845 to 1.242) | 0.805 |  | 45-54 | 0.771 (0.620 to 0.960) | **0.02** |
|  | 55-64 | 0.823 (0.757 to 0.896) | <0.001 |  | 55-64 | 0.756 (0.407 to 1.406) | 0.377 |
|  | 65-74 (reference) | 1 |  |  | 65-74 (reference) | 1 |  |
|  | primary | 1.077 (0.652 to 1.781) | 0.771 |  | primary | 2.413 (2.024 to 2.877) | **<0.001** |
|  | Secondary | 0.986 (0.771 to 1.261) | 0.908 |  | Secondary | 1.586 (1.495 to 1.683) | **<0.001** |
|  | Tertiary (reference) | 1 |  |  | Tertiary (reference) | 1 |  |
| Western | 25-34 | 1.891 (1.532 to 2.334) | <0.001 | **Western** | 25-34 | 1.971 (1.676 to 2.318) | **<0.001** |
|  | 35-44 | 1.516 (1.252 to 1.835) | <0.001 |  | 35-44 | 1.789 (1.625 to 1.970) | **<0.001** |
|  | 45-54 | 1.552 (1.331 to 1.809) | <0.001 |  | 45-54 | 1.551 (1.320 to 1.823) | **<0.001** |
|  | 55-64 | 1.224 (1.042 to 1.439) | 0.014 |  | 55-64 | 1.183 (0.889 to 1.575) | 0.249 |
|  | 65-74 (reference) | 1 |  |  | 65-74 (reference) | 1 |  |
|  | Primary | 1.677 (1.498 to 1.878) | <0.001 |  | Primary | 2.177 (1.653 to 2.868) | **<0.001** |
|  | Secondary | 1.202 (1.037 to 1.394) | 0.015 |  | Secondary | 1.342 (0.985 to 1.830) | 0.062 |
|  | Tertiary (reference) | 1 |  |  | Tertiary (reference) | 1 |  |

Table S8. Mixed-effects multinomial logistic regression model showing the association of frequency of alcohol consumption and socioeconomic variables, for aggregated 27 countries and per European subregions, without the interaction between sex and education.

| Variables | Adjusted OR (95%CI) | p value |
| --- | --- | --- |
| **European-wide**  **Female sex**  **Age**  25-34  35-44  45-54  55-64  65-74 (ref)  **Education**  Primary  Secondary  Tertiary (ref) | 2.82 (2.30 to 3.46)  1.42 (1.11 to 1.83)  1.30 (1.10 to 1.56)  1.12 (0.89 to 1.43)  0.99 (0.83 to 1.18)  1  1.74 (1.52 to 2.00)  1.29 (1.18 to 1.41)  1 | <0.001  0.006  0.003  0.337  0.911  <0.001  <0.001 |
| **Eastern Europe**  **Female sex**  **Age**  25-34  35-44  45-54  55-64  65-74 (ref)  **Education**  Primary  Secondary  Tertiary (ref) | 4.32 (3.76 to 4.95)  0.71 (0.56 to 0.90)  0.70 (0.61 to 0.81)  0.56 (0.41 to 0.76)  0.72 (0.66 to 0.78)  1  1.29 (1.03 to 1.62)  1.20 (1.00 to 1.43)  1 | <0.001  0.005  <0.001  <0.001  <0.001  0.027  0.045 |
| **Northern Europe**  **Female sex**  **Age**  25-34  35-44  45-54  55-64  65-74 (ref)  **Education**  Primary  Secondary  Tertiary (ref) | 2.22 (1.84 to 2.67)  2.64 (1.59 to 4.37)  2.07 (1.19 to 3.62)  1.16 (0.81 to 1.68)  1.12 (0.70 to 1.79)  1  2.75 (2.27 to 3.33)  1.97 (1.67 to 2.34)  1 | <0.001  <0.001  0.01  0.418  0.644  <0.001  <0.001 |
| **Southern Europe**  **Female sex**  **Age**  25-34  35-44  45-54  55-64  65-74 (ref)  **Education**  Primary  Secondary  Tertiary (ref) | 3.66 (3.01 to 4.46)  1.01 (0.78 to 1.30)  1.12 (0.86 to 1.44)  0.89 (0.79 to 1.00)  0.80 (0.57 to 1.14)  1  1.65 (1.21 to 2.27)  1.32 (1.15 to 1.53)  1 | <0.001  0.958  0.395  0.046  0.220  0.002  <0.001 |
| **Western Europe**  **Female sex**  **Age**  25-34  35-44  45-54  55-64  65-74 (ref)  **Education**  Primary  Secondary  Tertiary (ref) | 2.25 (1.76 to 2.89)  1.94 (1.70 to 2.22)  1.64 (1.46 to 1.85)  1.56 (1.38 to 1.76)  1.21 (1.06 to 1.38)  1  1.91 (1.62 to 2.25)  1.27 (1.10 to 1.47)  1 | <0.001  <0.001  <0.001  <0.001  0.005  <0.001  0.001 |

**Table S9.** Average Margin effects for the mixed-effects model on frequency of alcohol consumption and socioeconomic variables, per country.

| **Country** | **Female Sex (ref: male)** | |  |
| --- | --- | --- | --- |
|  | **Average** | **95% CI** | **p value** |
| **AT** | -0,154 | -0,186 to -0,122 | <0.001 |
| **BE** | -0,149 | -0,179 to -0,12 | <0.001 |
| **BG** | -0,155 | -0,186 to -0,123 | <0.001 |
| **CH** | -0,152 | -0,183 to -0,121 | <0.001 |
| **CY** | -0,153 | -0,183 to -0,123 | <0.001 |
| **DE** | -0,159 | -0,192 to -0,126 | <0.001 |
| **ES** | -0,155 | -0,186 to -0,123 | <0.001 |
| **FI** | -0,152 | -0,182 to -0,121 | <0.001 |
| **FR** | -0,157 | -0,189 to -0,125 | <0.001 |
| **GB** | -0,149 | -0,18 to -0,119 | <0.001 |
| **GR** | -0,155 | -0,186 to -0,124 | <0.001 |
| **HR** | -0,162 | -0,195 to -0,129 | <0.001 |
| **HU** | -0,156 | -0,189 to -0,124 | <0.001 |
| **IE** | -0,15 | -0,181 to -0,119 | <0.001 |
| **IS** | -0,143 | -0,174 to -0,112 | <0.001 |
| **IT** | -0,163 | -0,197 to -0,129 | <0.001 |
| **LT** | -0,151 | -0,182 to -0,121 | <0.001 |
| **LV** | -0,153 | -0,184 to -0,122 | <0.001 |
| **ME** | -0,154 | -0,187 to -0,121 | <0.001 |
| **NL** | -0,153 | -0,184 to -0,122 | <0.001 |
| **NO** | -0,148 | -0,178 to -0,118 | <0.001 |
| **PL** | -0,157 | -0,188 to -0,126 | <0.001 |
| **PT** | -0,158 | -0,191 to -0,125 | <0.001 |
| **RS** | -0,158 | -0,19 to -0,126 | <0.001 |
| **SE** | -0,149 | -0,179 to -0,119 | <0.001 |
| **SI** | -0,156 | -0,188 to -0,125 | <0.001 |
| **SK** | -0,154 | -0,186 to -0,122 | <0.001 |

| **Country** | **Age category 25-34 (ref:65-74)** | | | | **Age category 35-44 (ref:65-74)** | | | |  |
| --- | --- | --- | --- | --- | --- | --- | --- | --- | --- |
|  | **Average** | **95% CI** | | **p value** | | **Average** | **95% CI** | **p value** | |
| **AT** | -0,054 | -0,093 to -0,015 | | 0.006 | | -0,043 | -0,072 to -0,013 | 0.004 | |
| **BE** | -0,057 | -0,099 to -0,016 | | 0.007 | | -0,045 | -0,076 to -0,014 | 0.005 | |
| **BG** | -0,057 | -0,099 to -0,015 | | 0.007 | | -0,045 | -0,076 to -0,013 | 0.005 | |
| **CH** | -0,055 | -0,095 to -0,015 | | 0.007 | | -0,043 | -0,073 to -0,013 | 0.005 | |
| **CY** | -0,057 | -0,098 to -0,015 | | 0.007 | | -0,045 | -0,076 to -0,013 | 0.005 | |
| **DE** | -0,053 | -0,091 to -0,015 | | 0.006 | | -0,042 | -0,07 to -0,013 | 0.004 | |
| **ES** | -0,054 | -0,093 to -0,015 | | 0.006 | | -0,043 | -0,072 to -0,013 | 0.004 | |
| **FI** | -0,058 | -0,1 to -0,016 | | 0.007 | | -0,046 | -0,078 to -0,014 | 0.005 | |
| **FR** | -0,054 | -0,093 to -0,015 | | 0.007 | | -0,043 | -0,072 to -0,013 | 0.005 | |
| **GB** | -0,056 | -0,096 to -0,015 | | 0.007 | | -0,044 | -0,074 to -0,013 | 0.005 | |
| **GR** | -0,056 | -0,097 to -0,015 | | 0.007 | | -0,044 | -0,075 to -0,013 | 0.005 | |
| **HR** | -0,054 | -0,094 to -0,015 | | 0.007 | | -0,043 | -0,073 to -0,013 | 0.005 | |
| **HU** | -0,054 | -0,094 to -0,015 | | 0.007 | | -0,043 | -0,072 to -0,013 | 0.005 | |
| **IE** | -0,057 | -0,098 to -0,015 | | 0.007 | | -0,045 | -0,076 to -0,013 | 0.005 | |
| **IS** | -0,058 | -0,1 to -0,016 | | 0.007 | | -0,046 | -0,077 to -0,014 | 0.005 | |
| **IT** | -0,053 | -0,092 to -0,015 | | 0.007 | | -0,042 | -0,071 to -0,013 | 0.004 | |
| **LT** | -0,057 | -0,099 to -0,015 | | 0.008 | | -0,045 | -0,076 to -0,013 | 0.005 | |
| **LV** | -0,056 | -0,097 to -0,015 | | 0.008 | | -0,044 | -0,075 to -0,013 | 0.005 | |
| **ME** | -0,056 | -0,097 to -0,015 | | 0.007 | | -0,044 | -0,075 to -0,013 | 0.005 | |
| **NL** | -0,054 | -0,094 to -0,015 | | 0.006 | | -0,043 | -0,072 to -0,013 | 0.004 | |
| **NO** | -0,058 | -0,1 to -0,016 | | 0.007 | | -0,046 | -0,077 to -0,014 | 0.005 | |
| **PL** | -0,055 | -0,095 to -0,015 | | 0.007 | | -0,043 | -0,073 to -0,013 | 0.005 | |
| **PT** | -0,053 | -0,091 to -0,015 | | 0.007 | | -0,042 | -0,071 to -0,013 | 0.005 | |
| **RS** | -0,055 | -0,095 to -0,015 | | 0.007 | | -0,043 | -0,074 to -0,013 | 0.005 | |
| **SE** | -0,058 | -0,101 to -0,016 | | 0.007 | | -0,046 | -0,078 to -0,014 | 0.005 | |
| **SI** | -0,056 | -0,097 to -0,016 | | 0.007 | | -0,044 | -0,075 to -0,014 | 0.005 | |
| **SK** | -0,056 | -0,096 to -0,015 | | 0.007 | | -0,044 | -0,074 to -0,013 | 0.005 | |
|  | **Age category 45-54 (ref:65-74)** | | | | **Age category 55-64 (ref:65-74)** | | | |  |
| **Country** | **Average** | | **95% CI** | **p value** | | **Average** | **95% CI** | **p value** | |
| AT | -0,02 | | -0,058 to 0,018 | 0.299 | | 0,001 | -0,027 to 0,03 | 0.920 | |
| BE | -0,021 | | -0,061 to 0,019 | 0.300 | | 0,002 | -0,029 to 0,032 | 0.920 | |
| BG | -0,021 | | -0,061 to 0,019 | 0.301 | | 0,002 | -0,028 to 0,032 | 0.920 | |
| CH | -0,02 | | -0,059 to 0,018 | 0.300 | | 0,001 | -0,027 to 0,03 | 0.920 | |
| CY | -0,021 | | -0,061 to 0,019 | 0.301 | | 0,002 | -0,028 to 0,031 | 0.920 | |
| DE | -0,02 | | -0,057 to 0,017 | 0.299 | | 0,001 | -0,027 to 0,03 | 0.920 | |
| ES | -0,02 | | -0,058 to 0,018 | 0.299 | | 0,001 | -0,027 to 0,03 | 0.920 | |
| FI | -0,021 | | -0,062 to 0,019 | 0.301 | | 0,002 | -0,029 to 0,032 | 0.920 | |
| FR | -0,02 | | -0,058 to 0,018 | 0.300 | | 0,001 | -0,027 to 0,03 | 0.920 | |
| GB | -0,021 | | -0,059 to 0,018 | 0.301 | | 0,002 | -0,028 to 0,031 | 0.920 | |
| GR | -0,021 | | -0,06 to 0,019 | 0.301 | | 0,002 | -0,028 to 0,031 | 0.920 | |
| HR | -0,02 | | -0,058 to 0,018 | 0.300 | | 0,001 | -0,027 to 0,03 | 0.920 | |
| HU | -0,02 | | -0,058 to 0,018 | 0.300 | | 0,001 | -0,027 to 0,03 | 0.920 | |
| IE | -0,021 | | -0,06 to 0,019 | 0.301 | | 0,002 | -0,028 to 0,031 | 0.920 | |
| IS | -0,021 | | -0,062 to 0,019 | 0.299 | | 0,002 | -0,029 to 0,032 | 0.920 | |
| IT | -0,02 | | -0,057 to 0,018 | 0.300 | | 0,001 | -0,027 to 0,03 | 0.920 | |
| LT | -0,021 | | -0,061 to 0,019 | 0.302 | | 0,002 | -0,028 to 0,032 | 0.920 | |
| LV | -0,021 | | -0,06 to 0,019 | 0.302 | | 0,002 | -0,028 to 0,031 | 0.920 | |
| ME | -0,021 | | -0,06 to 0,019 | 0.301 | | 0,002 | -0,028 to 0,031 | 0.920 | |
| NL | -0,02 | | -0,058 to 0,018 | 0.299 | | 0,001 | -0,027 to 0,03 | 0.920 | |
| NO | -0,021 | | -0,062 to 0,019 | 0.299 | | 0,002 | -0,029 to 0,032 | 0.920 | |
| PL | -0,02 | | -0,059 to 0,018 | 0.299 | | 0,002 | -0,028 to 0,031 | 0.920 | |
| PT | -0,02 | | -0,057 to 0,017 | 0.300 | | 0,001 | -0,027 to 0,029 | 0.920 | |
| RS | -0,02 | | -0,059 to 0,018 | 0.301 | | 0,002 | -0,028 to 0,031 | 0.920 | |
| SE | -0,021 | | -0,062 to 0,019 | 0.300 | | 0,002 | -0,029 to 0,032 | 0.920 | |
| SI | -0,021 | | -0,06 to 0,019 | 0.300 | | 0,002 | -0,028 to 0,031 | 0.920 | |
| SK | -0,021 | | -0,059 to 0,018 | 0.301 | | 0,002 | -0,028 to 0,031 | 0.920 | |

| **Country** | **Primary Education (ref: Tertiary Education)** | | | **Secondary Education (ref: Tertiary Education)** | | | |  |
| --- | --- | --- | --- | --- | --- | --- | --- | --- |
|  | **Average** | **95% CI** | **p value** | | **Average** | **95% CI** | **p value** | |
| **AT** | -0,072 | -0,099 to -0,046 | <0.001 | | -0,034 | -0,049 to -0,018 | <0.001 | |
| **BE** | -0,073 | -0,1 to -0,046 | <0.001 | | -0,034 | -0,049 to -0,019 | <0.001 | |
| **BG** | -0,074 | -0,101 to -0,047 | <0.001 | | -0,035 | -0,05 to -0,019 | <0.001 | |
| **CH** | -0,073 | -0,1 to -0,047 | <0.001 | | -0,034 | -0,049 to -0,019 | <0.001 | |
| **CY** | -0,074 | -0,101 to -0,047 | <0.001 | | -0,035 | -0,05 to -0,019 | <0.001 | |
| **DE** | -0,074 | -0,101 to -0,047 | <0.001 | | -0,034 | -0,05 to -0,019 | <0.001 | |
| **ES** | -0,074 | -0,1 to -0,047 | <0.001 | | -0,034 | -0,05 to -0,019 | <0.001 | |
| **FI** | -0,073 | -0,1 to -0,046 | <0.001 | | -0,034 | -0,049 to -0,018 | <0.001 | |
| **FR** | -0,074 | -0,101 to -0,048 | <0.001 | | -0,035 | -0,05 to -0,019 | <0.001 | |
| **GB** | -0,073 | -0,099 to -0,047 | <0.001 | | -0,034 | -0,049 to -0,019 | <0.001 | |
| **GR** | -0,074 | -0,101 to -0,047 | <0.001 | | -0,034 | -0,05 to -0,019 | <0.001 | |
| **HR** | -0,075 | -0,103 to -0,047 | <0.001 | | -0,035 | -0,05 to -0,019 | <0.001 | |
| **HU** | -0,074 | -0,1 to -0,048 | <0.001 | | -0,035 | -0,05 to -0,019 | <0.001 | |
| **IE** | -0,072 | -0,098 to -0,046 | <0.001 | | -0,034 | -0,049 to -0,019 | <0.001 | |
| **IS** | -0,07 | -0,094 to -0,045 | <0.001 | | -0,032 | -0,046 to -0,018 | <0.001 | |
| **IT** | -0,075 | -0,102 to -0,047 | <0.001 | | -0,035 | -0,05 to -0,019 | <0.001 | |
| **LT** | -0,075 | -0,101 to -0,048 | <0.001 | | -0,035 | -0,051 to -0,02 | <0.001 | |
| **LV** | -0,075 | -0,102 to -0,049 | <0.001 | | -0,036 | -0,051 to -0,02 | <0.001 | |
| **ME** | -0,071 | -0,097 to -0,045 | <0.001 | | -0,033 | -0,048 to -0,018 | <0.001 | |
| **NL** | -0,073 | -0,1 to -0,046 | <0.001 | | -0,034 | -0,049 to -0,019 | <0.001 | |
| **NO** | -0,072 | -0,099 to -0,045 | <0.001 | | -0,033 | -0,048 to -0,018 | <0.001 | |
| **PL** | -0,075 | -0,103 to -0,047 | <0.001 | | -0,035 | -0,05 to -0,019 | <0.001 | |
| **PT** | -0,075 | -0,102 to -0,049 | <0.001 | | -0,035 | -0,051 to -0,02 | <0.001 | |
| **RS** | -0,074 | -0,101 to -0,047 | <0.001 | | -0,034 | -0,05 to -0,019 | <0.001 | |
| **SE** | -0,072 | -0,099 to -0,045 | <0.001 | | -0,033 | -0,048 to -0,018 | <0.001 | |
| **SI** | -0,073 | -0,101 to -0,046 | <0.001 | | -0,034 | -0,049 to -0,018 | <0.001 | |
| **SK** | -0,073 | -0,099 to -0,047 | <0.001 | | -0,034 | -0,049 to -0,019 | <0.001 | |

Figure S1. Marginal effects model for sex (reference: male), across 27 European countries.

Figure S2. Marginal effects model for age groups (reference ages: 65-74), across 27 European countries.

Figure S3. Marginal effects model for education (reference: tertiary education), across 27 European countries.
